# Testosterone promotes dominance behaviors in the Ultimatum Game after players’ status increases

**DOI:** 10.1038/s41598-023-45247-4

**Published:** 2023-10-21

**Authors:** Yukako Inoue, Robert P. Burriss, Toshikazu Hasegawa, Toko Kiyonari

**Affiliations:** 1 Department of Social Psychology, Yasuda Women’s University, Hiroshima, Japan; 2 Faculty of Psychology, University of Basel, Basel, Switzerland; 3 Graduate school of Arts and Science, The University of Tokyo, Tokyo, Japan; 4 School of Social Informatics, Aoyama Gakuin University, 5-10-1, Fuchinobe, Chuo-ku, Sagamihara, Kanagawa 252-5258 Japan

**Keywords:** Human behaviour, Social behaviour

## Abstract

Although testosterone is generally considered to promote dominance behaviors, in humans it fosters behaviors appropriate to achieving and maintaining social status, contingent upon the situation. Recent cross-sectional studies, such as Inoue et al. (Sci Rep 7:5335, 2017), have shown that dominance behaviors induced by testosterone are modulated by high status. Yet, it remains ambiguous whether a rise in social status within real-world social groups reshapes the relationship between testosterone and dominance behavior. To investigate this longitudinal question, we added a second wave to Inoue et al.’s study, collecting further data after an interval of 2 years. Members of a university rugby team that adheres to a rigid hierarchical order rooted in seniority played the Ultimatum Game with teammates and provided saliva for assays of testosterone and cortisol. Our analysis reveals that individuals with higher baseline salivary testosterone levels exhibited more dominance as their position in the hierarchy increased according to their seniority.

## Introduction

Testosterone (T) is a type of androgen and is associated with dominance behaviors, such as aggressive and competitive behaviors, both in various animals and in humans^1^. Studies have suggested that high T is positively associated with social (especially mating-related) aggression like male-male competition or territorial aggression, and that facing such competitions also promotes T secretion in various animals^2–6^. These results support the “challenge hypothesis”^7^.

Similar results have been found in humans (see reviews^1,8,9^), though the relationship between T and dominance is weaker than in other animals. A meta-analytic study revealed that there was only a “weak positive relationship” (Book et al.^10^, p. 579) between T and aggression in humans. This may be partly due to aggression or dominance being self-reported^8^. In contrast, economic games can measure dominance at the behavioral level. Economic games have been used in investigations of the effect of T on human dominance, but the results have been inconsistent. The present study aims to resolve this inconsistency by testing for the modulation effect of status. One of the economic games used for measuring dominance-seeking behaviors is the Ultimatum Game (UG)^11,12^. In the UG, one person is a “proposer” who makes an offer to a second, the “responder”, about how to divide a sum of money. This offer is an ultimatum: if the responder rejects it, neither receives any money. If the responder accepts the offer, then both receive money according to the proposer’s offer. Since receiving some money is better than nothing, any offer should be accepted from the viewpoint of maximizing self-interest. Contrary to this prediction, in market-integrated societies, small offers are frequently rejected by responders, and a substantial proportion of proposers tend to offer nearly half of the money^13,14^. Although this deviation from the maximization of self-interest has generally been considered to result from a desire for fairness, Burnham^12^ presented it in the context of a dominance challenge and showed that men with higher salivary baseline T were more likely to reject unfair offers in the UG.

Several studies using UG have shown that T promotes dominance behavior. For example, Mehta and Beer replicated Burnham’s result in a sample of men and women^15^. Zak et al. showed that the topical administration of T gel to male proposers resulted in them making lower offers^16^. Mehta et al.^17^ found that T also promotes competitive behavior in the hawk–dove game, a game that incorporates the concepts of victory and defeat^18^. Men and women who had higher baseline T tended to choose the competitive (hawk) option more frequently. However, there have also been results that do not fit this pattern. Studies using UG have reported that T administration did not affect the responders’ rejection^19,20^, or that T promoted generous behaviors, such as higher offers^21^ and lower rejections^22^.

What might explain this discrepancy? One possibility is that T has different effects depending on whether the situation represents a threat to status. This is supported by studies by Boksem et al.^23^ and Dreher et al.^24^. The effect of T on behaviors in the Trust Game (TG)^25^ was examined by Boksem et al. The TG is played by an “investor” and a “trustee.” Boksem et al. found that T administration in women was associated with investors transferring less money to trustees but trustees returning more money to investors. The TG allows for an investor’s trust to be betrayed by the trustee, and such betrayal may be interpreted by the investor as a threat to their status^24^. T may therefore inhibit an investor from investing larger amounts, thereby denying the trustee an opportunity to betray. On the other hand, trustees face no equivalent threat to their status because they are the final decision makers. In such situations, T appears to promote altruistic behaviors rather than dominance behaviors.

Dreher et al. used a modified UG incorporating punishment and reward and reported that administering T to male responders resulted in their punishing more strongly proposers who had offered small amounts, but also rewarding more generously proposers who had offered large amounts, perhaps because low rather than high offers caused responders to feel a threat to status^25^.

These results suggest that T promotes dominance behavior when a person feels a threat to their status, and generous behavior when a person feels their status is respected. This may be because dominance behaviors only serve to guard and promote status when status is under threat. In the absence of a threat to status, dominance behaviors may decrease status, whereas generous behavior is likely to enhance status. In summary, as Eisenegger et al. has stated, T may promote “the motivation to achieve and maintain high status” in humans (Eisenegger et al.^9^, p. 265, Figure 1): whether that motivation leads to dominance behavior or generous behavior depends on the situation. Possible discrepancies between studies in participants’ perception of threat to status may also explain the weak positive correlation that has been observed between T and dominance behavior in economic games. Asymmetrical economic games, such as the UG, are associated with differences in the players’ subjective status (i.e. which role has more power), which may cause differences in the players’ perception of threat to status.

Consistent with this explanation, some recent studies have found that status and self-reported dominance may modulate the effects of T on dominance behaviors. Some studies have suggested that T promotes competitive or dominance behaviors only in individuals with high trait dominance^26^ or higher experimentally manipulated status than the opponent^27^ (for a review see Carré & Archer^28^). Furthermore, there are two studies, to our knowledge, that investigate the effect of objective status in a real-world social group, which represents a design with higher ecological validity. One is Siart et al.^29^ In this laboratory-based study, high- and low-ranking soldiers were paired and allocated guard duty, with one member of each pair randomly assigned to stand guard and the other to rest. The soldier in the guard role proposed a period of time that they would stand guard. The resting soldier could reject the offer, in which case the guard made further offers until the resting soldier agreed. The period of duty then began. After the period ended, the resting soldier decided whether to switch roles. The task was then repeated until a total of 40 min of guard duty had elapsed. In this study, the correlation between salivary T levels and the duty allocation was not significant. In addition, there was no modulation effect of rank. However, this study is limited in several respects. First, the guard duty—standing in front of a computer—may not have been obvious to participants, who may have responded differently than they would have with more obvious incentives. Second, the study used deception to investigate the behavior of participants being treated unfairly, although it may have been possible for participants to learn of the purpose of the experiment due to their existing social connections. There were two conditions: non-deceptive and deceptive. In the deceptive condition participants were first assigned to stand guard and the ostensibly real partner (actually a computer) was programmed never to switch roles, whereas in the non-deceptive condition participants interacted with real partners. Since the participants were recruited from the same barracks, participants in the deceptive condition could have communicated about the experiment to later participants, possibly sowing doubt about the reality of very unfair partners.

The other study, by Inoue et al., investigated the effect of status on the relationship between T and dominance behaviors in a real-world social group using economic games with a monetary incentive^30^. This study conducted an experiment with a real-world social group of participants—members of a Japanese university rugby team—whose hierarchical relationships are strictly based on their seniority. The inherent tie between seniority and hierarchical position makes a Japanese university sports team an ideal sample for probing the modulation effect of status rather than the influence of an individual’s personality traits on obtaining status. Baseline T and dominance behavior were positively related only in the most senior members: those who are most likely to have felt their status threatened by unfair offers. There was a negative relationship between T and dominance behavior in other lower-ranking members.

No study has investigated whether shifts in social status within real-world social groups lead to differences in the relationship between T and dominance behavior. We therefore supplemented the cross-sectional data collected by Inoue et al.^30^ by administering to many of the same participants a second wave of tasks after an interval of two years. By adding a longitudinal component to the dataset, we were able to examine the impact of rising status on the relationship between T and dominance behavior.

We also investigate the effect of cortisol (C), the glucocorticoid hormone released in response to stress, which may modulate the relationship between T and dominance behaviors^31^. Because C inhibits the neuroendocrine system regulating T, and high C is related to high stress and social avoidance, high T leads to high motivation for social status when C is low^32^. This interaction between T and C is found in studies in which dominance is measured by self-report^33^, evaluated by a third party^31,34^, and assessed as behavior in certain economic games^35,36^. For example, Pfattheicher et al. found that higher baseline T is associated with stronger antisocial punishment (the punishment of cooperators) in public goods games, but only in individuals with low C^35^. In another study Pfattheicher found that self-reported trait dominance modulated the interaction between T and C: although T was related to dominance behavior in the adapted dictator game in individuals with low C levels, this tendency was seen only in participants with high self-reported dominance^36^. Therefore, it is important to confirm this second-order interaction between T, C, and dominance using objective status measures rather than self-reported dominance.

## Methods

We conducted an experiment two years after the study by Inoue et al.^30^, recruiting members of the same university rugby team. We refer to the data collected by Inoue et al.^30^ as the “first wave” and data collected two years later as the “second wave.” We combine the data from both waves for our longitudinal analysis. Half of the students who participated in the first wave had graduated by the time of the second wave. Those who were in the first- or second-year during the first wave were third- or fourth-year students at the time of the second wave. The methodology employed in the first wave has been described in detail in Inoue et al.^30^, so here we only describe the methodology of the second wave.

### Participants

We recruited 71 male undergraduate members, aged between 18 and 23 years, of Aoyama Gakuin University’s rugby team. The participants included 21 first-year students, 18 second-year students, 13 third-year students, and 19 fourth-year students. Thirty of these students (12 third-year and 18 fourth-year) had participated in the first wave.

### Study protocol

We conducted the study in a large conference room. We collected saliva samples in the morning (9:45–10:45 a.m.), as close as possible to the collection time in the first wave. Due to the team’s regular practice schedule, the UG task was conducted in the afternoon (3:30–4:00 p.m.). Following the UG task, we collected saliva samples again, and participants completed a questionnaire. Although we collected post-experiment saliva samples, we do not report the analysis of those samples here because we cannot eliminate the influence of practice on T changes. Please see Supplementary Information 4 for details of this analysis.

Participants received a monetary reward that included a show-up fee of JPY 1000 and a supplementary reward according to the result of the UG. Participants received their show-up fee on the day of the experiment and the supplementary reward later, after the results had been calculated. They were informed about these payment protocols in advance.

**Figure 1:**
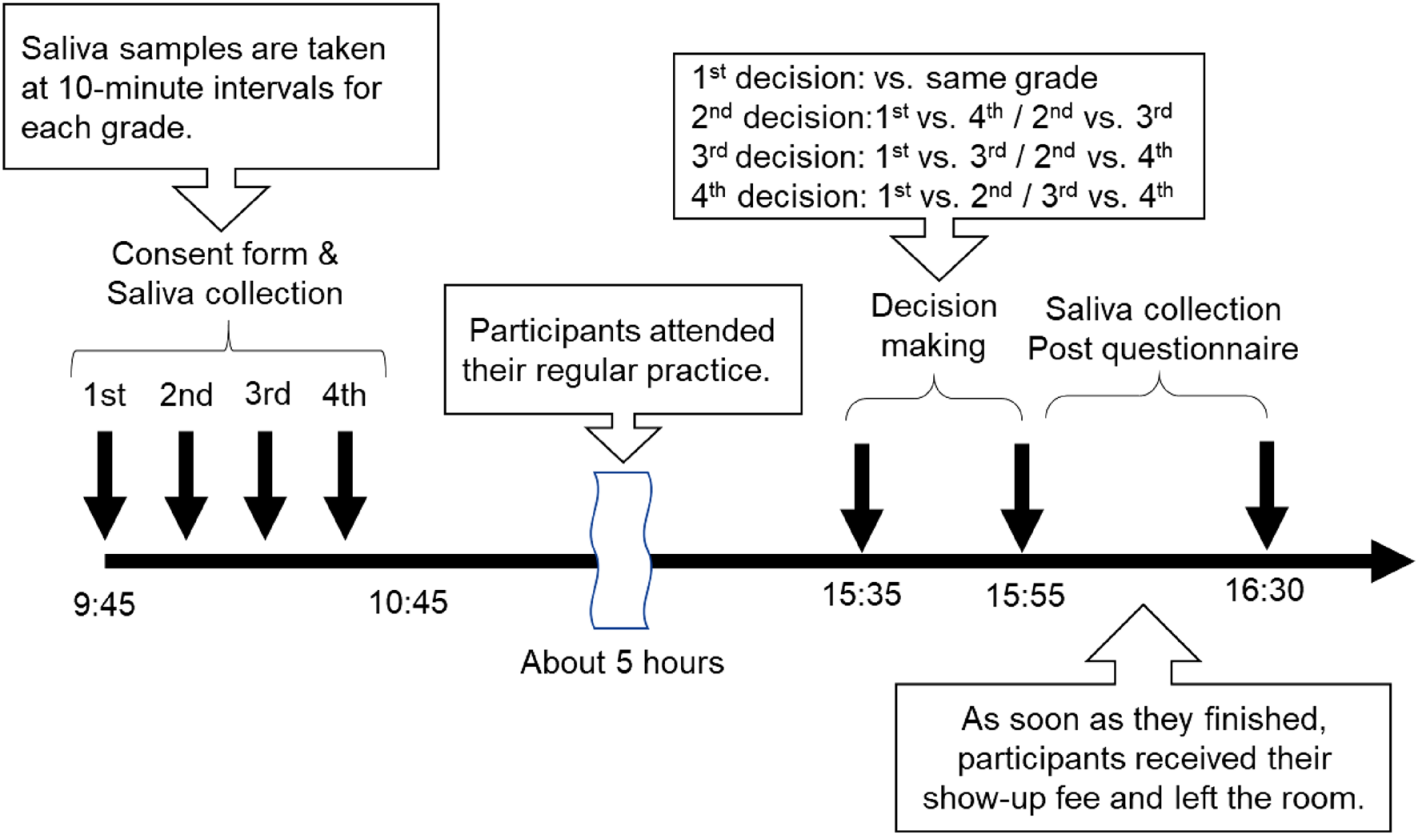
A summary of the experimental protocol.

The study protocol is illustrated in Fig. 1. The protocol was approved by the Ethics Committee of Aoyama Gakuin University, and met the requirements of the Declaration of Helsinki. All participants gave written informed consent before saliva collection.

### Saliva collection

Participants were told in advance to avoid drinking alcohol, smoking, and taking caffeine on the day before and the day of the experiment. Male experimenters collected participants’ saliva in the morning. Participants came to the conference room in groups according to their year, starting with the first years. Participants delivered 5 ml of saliva into a cryotube via passive drool. Saliva specimens were immediately frozen in a cooler box full of dry ice. After all tasks were completed, the specimens were stored at −80 °C and later assayed in LC–MS/MS with outsourcing to ASKA Pharma Medical Co.

### One-shot ultimatum game (UG)

The procedure of the UG in the second wave was identical to that in the first wave, except in the method of pair matching. Participants first received instructions about the rules of the UG. They were informed that they would play the UG four times, each time with a different opponent. Each game would consist of two stages, and the participant would play one stage as a proposer and one as a responder.

Participants competed against an opponent from each year, including from their own year. The order of play is described in Fig. 1. In the first wave, participants played four different games. The first was the “no information game,” where participants played without any information about their opponents’ seniority. The second was the “peer-to-peer game,” where participants played against opponents from the same year. The third was the “fourth-year game,” where fourth-year students played against junior opponents (first to third years). The fourth type was the “first-year game,” where first-year students played against senior opponents (second to fourth years). However, for the third and fourth types, the difference in academic years between the players and their opponents depended on their own year. For example, in the “fourth-year game,” combinations like fourth-year vs. first-year, fourth-year vs. second-year, fourth-year vs. third-year were possible. In these cases, the difference in seniority between the fourth-year students and their opponents varied. Participants who believed they were competing against first-year students might have reacted differently to those who believed they were facing third-year students. Therefore, in the second wave, to remove this ambiguity regarding the presumed status of the opponent, we adjusted the design so that participants competed against opponents from each year, including those from the same year.

Participants were further informed that they would receive a supplement to their show-up fee based on their responses during a randomly selected two of the four games. We ensured that participants fully understood the procedure for calculating the supplementary reward. The supplementary reward was based on the results of two randomly selected games of the four total games, and the value of the reward corresponded to the real values specified in the games. While playing the games, participants indicated their choices in a booklet, which they hid from the view of the other participants using a cardboard fence. We refer the reader to the Supplementary Information 6 for further details of the instructions given to participants.

Acting as proposer, each participant decided how to divide a monetary endowment between himself and a matched anonymous responder. Proposers decided upon a sum to offer the responder that ranged between nothing and JPY 1000, at increments of JPY 100. As the responder, participants indicated whether they would accept or reject the matched proposer’s offer if it was at each of the 11 possible levels (ranging from JPY 1000 for the proposer and nothing for the responder, to nothing for the proposer and JPY 1000 for the responder). The responder was not made aware of the actual offer made by the proposer. This method of measuring the responder’s choices is termed the strategy method. If the responder rejects at the level corresponding to the actual offer made by the proposer, both the proposer and the responder receive no money. If the responder accepts at the actual offer level, the players receive funds as proposed. Because the number of participants in each year differed, forming one-to-one pairs across each year was impossible. Therefore, participants in year groups with smaller numbers were matched with more than one opponent. In these cases payment calculations were based on one randomly selected result.

The main dependent variable is the degree of “acquiescence”^30^, calculated as the difference between offers made by players in the proposer role and the offers those same players tolerate as the responder. Prior to calculating acquiescence, we calculated the minimum acceptable offer (MAO) for each participant based on their responses to the 11 possible levels. If participants showed a non-linear response (for example, rejecting an offer of JPY 100, accepting an offer of JPY 200, but rejecting an offer of JPY 300), we omitted such responses from the corresponding analyses because we could not calculate MAO. Then we calculated acquiescence as the value of the offer minus MAO. Acquiescence has been called “generosity” in another study^16^, but we consider “acquiescence” more appropriate when players differ in rank. This is because accepting a lower offer in such a situation reflects a willingness to yield to the other player’s implicit coercion rather than the desire to give the proposer more. A lower (especially negative value) acquiescence indicates dominance behavior because such a participant requires more as a responder than they give others as a proposer.

### Analyses

The data relating to salivary hormones (T and C) were log-transformed before the statistical analyses because they were not normally distributed. We first report on the analysis of the data from the second wave and then on the analysis of longitudinal data.

First, we analyzed the data of the second wave alone for comparison with the first wave’s result. In this analysis, we classified all decisions into three conditions: (1) the senior opponent condition; (2) the same-year opponent condition; and (3) the junior opponent condition. This is because we expected that decisions would be affected by whether the opponent has a higher or lower seniority (status) than the decision-maker, rather than the opponent’s year itself. We averaged the behavioral indexes for each condition. Note that the first years did not experience the junior opponent condition because there were no participants junior to them, and the fourth years did not experience the senior opponent condition because no participants were senior to them. The behavioral indexes of these conditions were treated as missing values. To investigate the effect of status, we analyzed acquiescence by GLMM with fixed effects of seniority, the opponent conditions, pre-measured T, and each interaction. The random effect was participants. Then, we conducted GLMM, adding pre-measured C and relevant interactions as fixed variables in order to investigate the modulation effect of C. Prior to these analyses, T and C were centered to facilitate the interpretation of estimated coefficients.

In the analysis of the longitudinal data, we included the data of the first and second waves. We used the averages of behavioral indexes among all opponent conditions as the dependent variables and did not include the opponent conditions as the fixed effect. This is because both years have missing values in either the senior opponent condition or junior opponent condition: those who were the first-year students in the first wave have a missing value for the junior opponent condition at that time, and those who were the second-year students in the first wave have a missing value for the senior opponent condition in the second wave, when they were fourth-year students.

We conducted the analysis of mean acquiescence in all conditions by GLMM. The fixed effects were participants’ years, the wave (first/second), pre-measured T, and each interaction. The random effect was participants. We conducted a further GLMM, adding pre-measured C and relevant interactions as fixed variables. In these analyses, hormones were standardized for each study.

The behavioral indexes did not normally distribute, so we adopted the lognormal distribution in all GLMMs because this model had the lowest Akaike’s Information Criterion (AIC) among some continuous probability distribution (normal distribution, t-distribution, gamma distribution, and lognormal distribution). To prepare for this analysis, we added 1000 to acquiescence because all values must be positive in a GLMM using lognormal distribution. We used the glimmix procedure of SAS 9.4 in these analyses. We set the link function as identity. The parameters were estimated using the Laplace approximation. We selected variables by backward selection and chose the best model based on AIC.

We also conducted GLMM in the same way with other behavioral indexes (MAO and the offer) as dependent variables. In addition, we assayed C from first wave samples (ASKA Pharma Medical Co.) and reanalyzed it using GLMM. These results are reported in the supplementary materials.

## Results

First, we analyzed the data of the second wave alone. The descriptive statistics of pre-game measures of hormones (Table S1) and behavioral indexes (Table S2) are shown in the Supplementary Information 1. With regard to hormones, there was a significant difference in pre-game C as a function of participants’ seniority (*F*(3, 67) = 5.25, *p* < 0.01, η_p_^2^ = 0.190). According to the multiple comparisons, the pre-game C of first-year students was lower than that of the other three groups. There was no difference in T as a function of seniority (*F*(3, 67) = 0.39, *p* = 0.76, η_p_^2^ = 0.017). The correlation between pre-game T and pre-game C was positive but not significant (*r*_*s*_ = 0.11, *p* = 0.35). With regard to behaviors, it appears that students earlier in their academic career were more acquiescent (see Table S2). These results were consistent with the first wave (see Supplementary Information 5), except for the significant positive correlation between pre-game T and C. However, given that most previous studies have reported positive correlations (e.g. Ref.^31,33,34,36^) or non-significant correlations (e.g. Ref.^34,35^) between T and C, these results suggested baseline T and C have a weak positive relationship, whether or not it reaches the conventional significance level.

**Figure 2:**
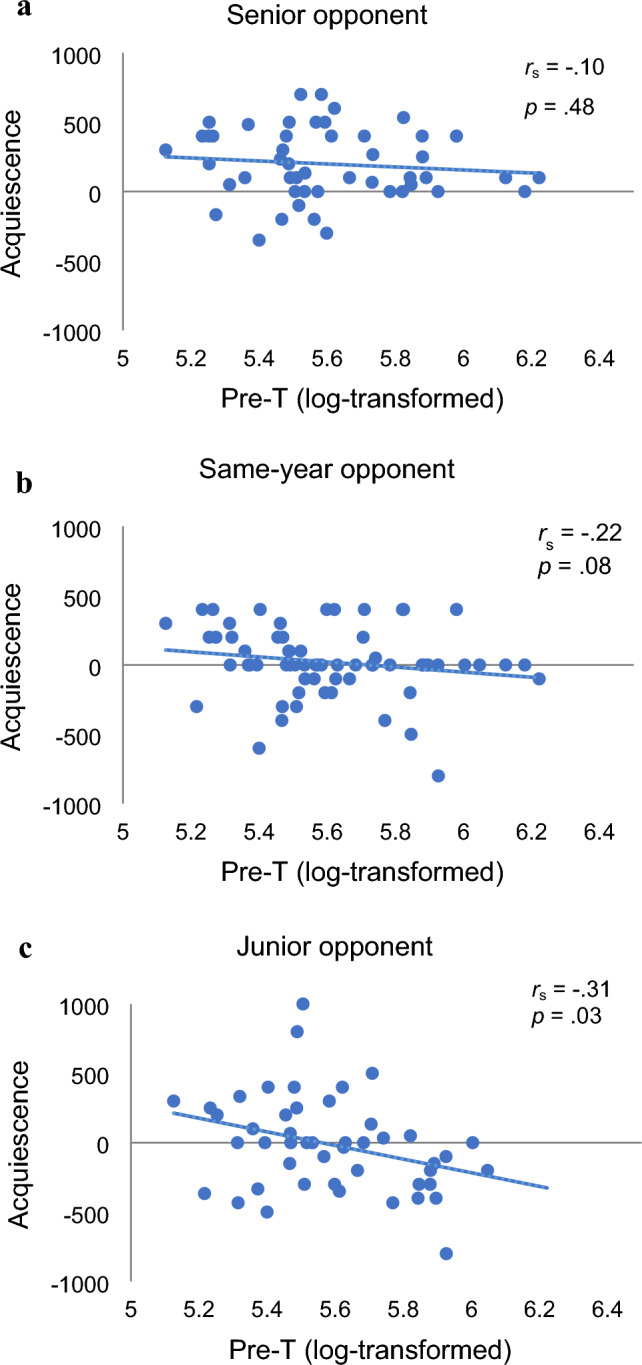
The scatter plot between pre-game T level and acquiescence. (**a**) Senior opponent condition, (**b**) Same-year opponent condition, (**c**) Junior opponent condition.

We analyzed acquiescence in the second wave by GLMM with fixed effects of seniority, the opponent condition, and T and random effect of participants. As a result, the model including only T, opponent condition, and their interaction was adopted as the best model when the junior opponent condition was set as the contrasts (Table S3). There was a significant difference between the senior opponent condition and the junior opponent condition: participants acquiesced more when their opponent was senior rather than junior. In addition, the interaction of opponent condition and T was significant: the estimated coefficient of T was at its lowest in the junior opponent condition. As shown in Fig. 2, in the junior opponent condition, salivary T showed a significant negative correlation with acquiescence. However, there were non-significant negative correlations in the other two conditions. This is a different result from the first wave, where baseline T and acquiescence were positively correlated among the lower year students.

**Figure 3:**
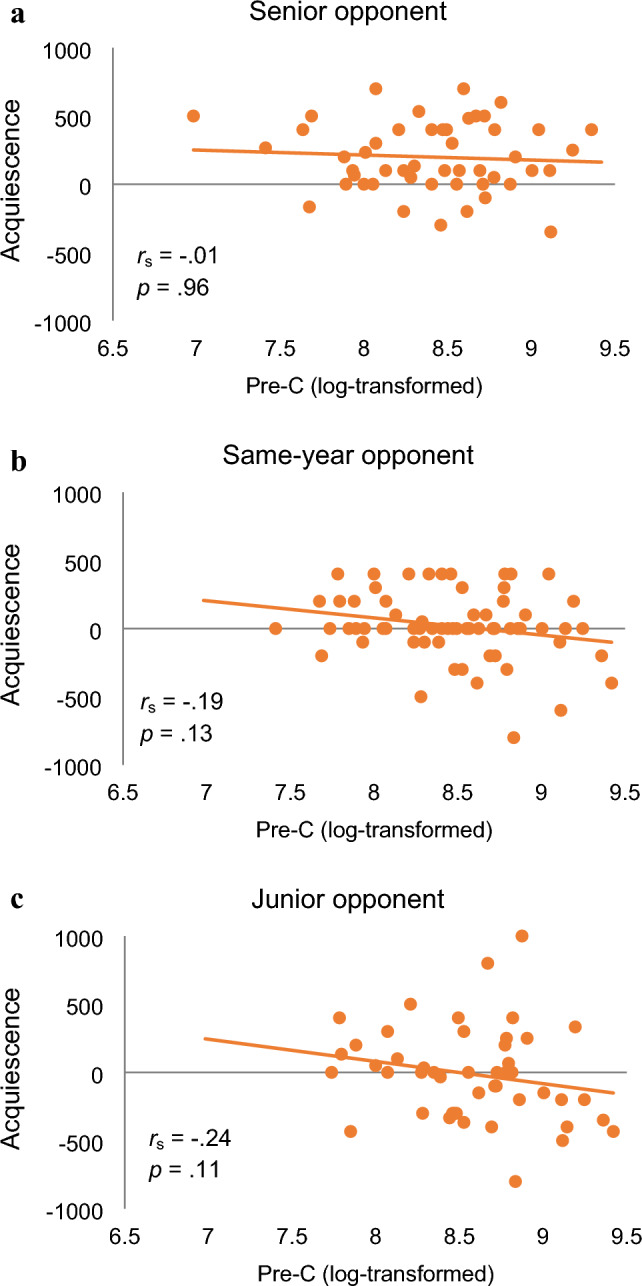
The scatter plot between pre-game C level and acquiescence. (**a**) Senior opponent condition, (**b**) Same-year opponent condition, (**c**) Junior opponent condition.

This tendency was also maintained when C and relevant interactions were added as a fixed effect (Table S4). Adding the fixed effect of C to the model of Table S3 resulted in the best model. In this model, there was a significant effect of opponent condition between senior and junior opponent conditions. There was also a significant interaction of T and opponent condition between the senior and junior opponent conditions, and a marginally significant interaction between same-year opponent condition and junior opponent condition. In addition, the main effect of C was marginally significant. However, the correlation between C and the level of acquiescence was negative but non-significant in all opponent conditions (Fig. 3).

Next, we combined data from the first and second waves to investigate the effect of increasing seniority on behavior and hormones. The descriptive statistics of pre-game measures of hormones and behavioral indexes in participants who participated in both waves are shown in Tables S5 and S6. First, we calculated the correlation between pre-transformed hormones measured in both waves. As a result, participants’ pre-measured T was positively correlated between both waves (*r*_*s*_ = 0.42, *p* = 0.02). However, C was not significantly correlated (*r*_*s*_ = 0.05, *p* = 0.79). Next, we calculated the average of the hormone changes between both waves (Table S5). We found no statistically significant change in the baseline levels of T (Wilcoxon signed-rank test: S = 30, *p* = 0.55). However, the baseline of C was significantly increased (Wilcoxon signed-rank test: S = 171, *p* < 0.001).

Similarly, we calculated the correlation and the change of behavioral indexes between both waves. Participants’ acquiescence was significantly positively correlated (*r*_*s*_ = 0.42, *p* = 0.03), although the correlation between the other two indexes were not significant (MAO: *r*_*s*_ = 0.27, *p* = 0.17, offer:* r*_*s*_ = 0.05, *p* = 0.81). As with the changes, all behavioral indexes showed that participants behaved more dominantly in the second wave than in the first wave (Table S6). In other words, MAO in the second wave was higher than in the first wave (Wilcoxon signed-rank test: S = 64.5, *p* = 0.047), and both the offer and acquiescence in the second wave tended to be lower than in the first wave (Wilcoxon signed-rank test; the offer: S = −103, *p* < 0.01; the acquiescence: S = −118, *p* < 0.001).

**Figure 4:**
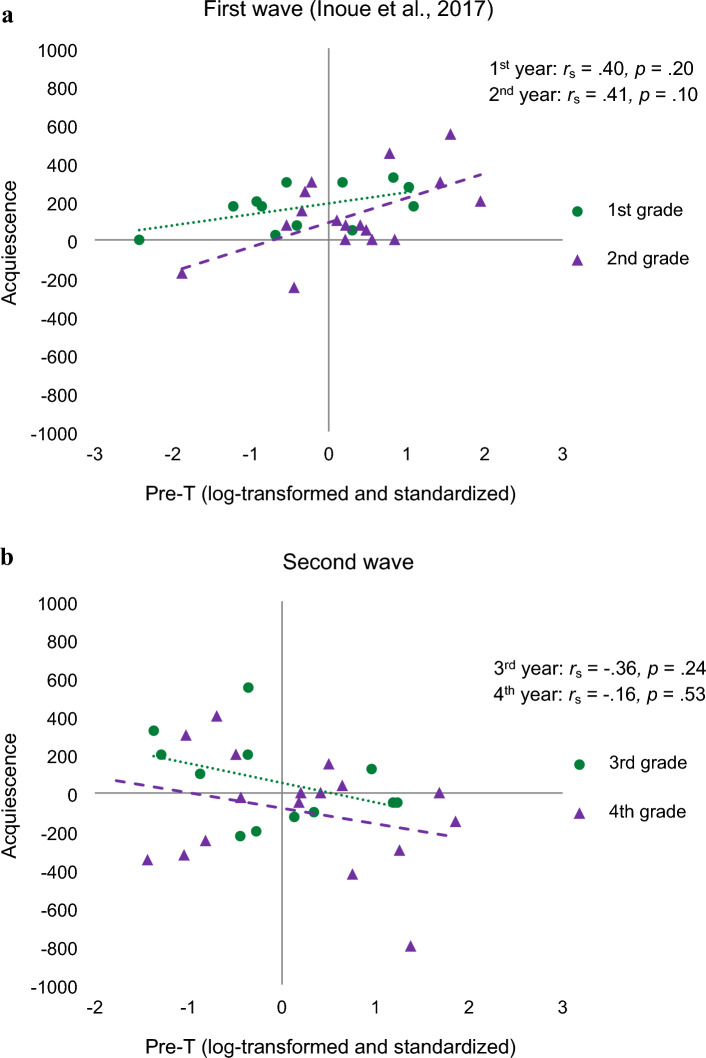
The scatter plot between pre-game T level and acquiescence in each wave. (**a**) first wave, (**b**) second wave.

We analyzed acquiescence by GLMM with fixed effects of participants’ seniority, the wave (first/second), and T and random effect of participants (Table S7). As a result, the best model contained each main effect and the interaction between T and wave. Note that the covariance parameter estimate of the random effect was zero in this model. Although the random effects were left in the model based on the experimental design, the model estimations were the same as those without the random effects (cf.^37^). In this model, the main effect of the study was significant; participants came to behave more dominantly in the second wave. The difference between seniority and the effect of T in contrasts were marginally significant. Most importantly, the interaction between T and wave was significant. Although participants with high baseline T were more inclined to acquiesce in the first wave when their status was lower, this tendency was reversed in the second wave when their status was higher (Fig. 4).

These effects were also significant (or marginally significant) when C and relevant interactions were added as a fixed effect. The best model is shown in Table S8. Again, the covariance parameter estimate of the random effect was zero in this model. Although there remained some C-relevant effects in the best model, none of the effects was significant.

## Discussion

Our study in a real-world hierarchical group revealed that endogenous T is related to dominance behaviors depending on social status. First, we analyzed the data of the second wave and compared it with the first wave^30^. In both waves, the higher a participant’s endogenous T, the lower his acquiescence (he behaved more dominantly when he was senior to his opponent). This suggests the robustness of the result that high endogenous T promotes dominance behaviors only in relatively high-status people. Similar modulations of status or higher trait dominance on the effect of T has been reported in some studies, as described in the introduction^26,27^. On the other hand, there were non-significant but negative relationships between endogenous T and acquiescence when the player was equal or junior to their opponent in the second wave. This result is inconsistent with the first wave, where higher endogenous T was associated with higher acquiescence in junior players. Considering that T promotes “strategic” behavior to achieve high status rather than dominance behavior per se, testosterone might encourage submissive behavior if it is effective in securing high status^9^. Within Japanese university sports teams, which operate under a strict hierarchy, individuals ascend the hierarchy solely by advancing in seniority. In such a situation, dominant or confrontational behavior might hinder one’s pursuit of elevated status. According to studies of mafias, which have similar self-governing organizational structures, one of the fundamental rules shared across various mafias is “Do not disobey, or cause a nuisance to your superiors” (Catino^38^, p. 539), which plays a crucial role in maintaining order within the mafia organization. That aggressive members show obedience to their superiors under certain circumstances might not be surprising. It remains unknown whether the relationship between baseline T and submissive behavior among low-status members was false positive or whether there are some other modulating factors (i.e. the interpretation of the anonymous UG situation). This point would be worth further investigation.

Next, we investigated the shift in the effect of testosterone as a function of rising status by conducting longitudinal analyses of data from the two waves. The negative relationship between endogenous T and acquiescence strengthened as participants advanced in their academic careers. This finding indicates that within the same individuals, the relationship between high baseline testosterone and dominance behavior alters in response to their status within the group. Specifically, a higher baseline T leads to more dominant behavior as status increase.

Among the three behavioral indexes, only acquiescence showed this tendency. MAO and offer showed inconsistent relationships with endogenous T (see Supplementary Information 2). As outlined by Inoue et al.^30^, these indexes may not be valid measures of dominance behavior compared to acquiescence. The low correlation coefficients of these two indexes in the longitudinal analysis may also support this conclusion.

In addition, it is important to note that the influence of hormones on dominance behavior should not be overestimated. Participants showed more dominant behavior when their status was higher than their opponent’s. Generally MAO was higher and both offer and acquiescence were lower when a participant was in years 3 or 4, or when the opponent was junior to the participant.

We also investigated the interaction between T and C (dual-hormone hypothesis^31^) and the effect of C on the interaction between T and status. We found no evidence for the dual-hormone hypothesis: in no analysis was the interaction between T and C significant. The interaction between T and C may be affected by various other factors. Recently, a meta-analysis found that although the T and C interaction effect was significant, its effect size was very small, and the variance of effect sizes was large, and so provided only marginal support for the dual-hormone hypothesis^39^. Knight et al.^40^ discussed the cause of this instability of the dual-hormone hypothesis. They proposed some possible modulation factors, including the social cues of status and the threat of status. We must note, however, that the second-order interaction was not significant: C did not affect the interaction between T and status. Instead, the interaction between T and status remained significant or marginally significant when we added C as the independent variable. This result may indicate that the modulation effect of status on the relationship between baseline T and dominance behavior was robust when considering C. However, Pfattheicher reports a second-order interaction between T, C, and status^36^. The modulation effect of status in tests of the dual-hormone hypothesis should be a focus of future research.

The following three points, which were not the main topics of this study, may also be interesting topics for the future. The first topic is the relationship between status and baseline cortisol. In our study, the baseline cortisol of the first-year students was consistently lower than in other students. The relationship between cortisol and real status in real-world social groups in humans is not well understood. In animals, the relationship between baseline cortisol and status depends on whether occupying a higher or lower status is more stressful. The comparative study of primates shows that low-status individuals have higher C than high-status individuals in species with higher stress or less social support for low-status individuals^41^. Although most studies on cortisol and status in humans define status in socio-economic terms, a review by Dowd et al. argues that socio-economic status is not consistently associated with baseline cortisol^42^. One of the few studies measuring endogenous C in a real-world hierarchical group is Siart et al.^29^. In this study, salivary C measured before the duty allocation task was higher in low- than in high-ranking soldiers. But, as participants were informed before providing saliva that they would play the allocation task with a partner of different rank, it is questionable whether a pure baseline C was measured. Studies measuring baseline C in real-world hierarchical groups are still scarce, and the effect of an individual’s social status on baseline C is unknown. Further investigation is needed.

The second topic is the relationship between acute hormone changes and dominance behaviors. While our study focused on the effect of baseline T on dominance behavior, recent studies have paid more attention to the changes in T when facing provocations or competitions. Carré and Archer^28^ state that “acute changes in testosterone within the context of competition and/or social provocation may be more relevant for understanding individual differences” (p. 150) than baseline T. Nevertheless, we did not design our experiment such that changes in T could be analyzed. One reason for this is that the changes in T did not affect dominance behaviors in Inoue et al.^30^ (see Tables S9, S11, S13). Another reason, and perhaps the most critical procedural issue, is that we were obliged to conduct the experiment around the rugby team’s regular practice due to their schedule constraints. In regard to hormone changes before and after the economic games, future studies are needed.

The third topic is the effect of testosterone in women. We wanted to investigate the effect of position in a real-world social hierarchy in those engaged in contact sports. Given that only a men’s rugby team was within our accessible range, our study was limited to men. Studies examining testosterone effects in samples of women and hierarchical governance in women’s sports teams are scarce, so investigating T-relevant effects in women is imperative^43^. This is an issue for future consideration.

In summary, our study demonstrated the context-dependency of the relationship between testosterone and dominance behavior, in line with Slatcher et al.^26^ and Mehta et al.^27^. The study by Inoue et al. was the first to show that status in actual real-world social groups modulates this relationship: people with high baseline testosterone tend to behave more dominantly only when their status is high. Here, in a longitudinal extension to that study, we show that those with higher baseline testosterone display more dominance as their status increases. The implications of this research for the study of dominance and status lie in the complexity of achieving or maintaining high status. In humans, the relationship between hormones and behaviors may be determined by various factors—current status, the presence of threat to status, and effective or available ways to achieve high status (cf. dominance and prestige^44–46^). Therefore, attention must be paid to these factors when investigating the relationship between hormones and behaviors, especially in abstract situations like economic games.

### Supplementary Information

Supplementary Information.

## Data Availability

The datasets cords generated during and/or analyzed during the current study are available in the open science framework (https://osf.io/z7qau/).
