# Supplementary material for: Testosterone promotes dominance behaviors in the Ultimatum Game after players’ status increases
Source: Sci Rep. 2023 Oct 21;13:18029. doi: 10.1038/s41598-023-45247-4 (PMC10590433; doi:10.1038/s41598-023-45247-4)
Supplement: Supplementary file 1 — Supplementary Information. [file 41598_2023_45247_MOESM1_ESM.pdf]

## Supplementary Information 1. Tables and Figures related to the main text

Table S1. The value of pre-measured hormones

| | | original value (pmol / L) | | Log-transformed | |
|---|---|---|---|---|---|
| | N | T | C | T | C |
| 1st year | 21 | 295.9 (95.2) | 3616.9 (1663.8) | 5.64 (0.30) | 8.09 (0.47) |
| 2nd year | 18 | 273.4 (56.8) | 5415.3 (2100.8) | 5.59 (0.21) | 8.53 (0.38) |
| 3rd year | 13 | 263.5 (58.0) | 6061.3 (2546.3) | 5.55 (0.22) | 8.63 (0.43) |
| 4th year | 19 | 275.7 (75.7) | 5618.2 (2733.2) | 5.58 (0.27) | 8.52 (0.49) |
| All | 71 | 278.8 (74.6) | 5056.0 (2413.1) | 5.60 (0.25) | 8.42 (0.49) |

*Note*. SD is in parentheses.

Table S2. Means and standard deviations of behavior indexes of UG

| | MAO | | | |
|---|---|---|---|---|
| | Senior opponent | Same-year opponent | Junior opponent | Total |
| 1st year (N=20) | 286.67 (183.02) | 394.44 (155.19) [a] | — | 312.50 (162.93) |
| 2nd year (N=16) | 275.00 (178.89) | 275.00 (191.49) | 281.25 (207.26) | 276.56 (169.67) |
| 3rd year (N=13) | 246.15 (145.00) | 361.54 (160.93) | 353.85 (207.63) | 328.85 (166.10) |
| 4th year (N=18) | — | 372.22 (180.87) | 387.04 (194.08) | 383.33 (185.70) |
| Total | 272.11 (169.67) | 352.31 (175.10) | 341.84 (203.20) | 326.12 (172.13) |
| | **Offer** | | | |
| | Senior opponent | Same-year opponent | Junior opponent | Total |
| 1st year (N=21) | 530.95 (154.05) | 476.19 (137.49) | — | 517.26 (126.77) |
| 2nd year (N=18) | 477.78 (196.46) | 316.67 (191.74) | 338.89 (247.67) | 402.78 (161.99) |
| 3rd year (N=13) | 415.38 (181.87) | 369.23 (154.84) | 369.23 (182.05) | 380.77 (141.48) |
| 4th year (N=19) | — | 313.16 (188.45) | 278.95 (178.89) | 287.50 (169.71) |
| Total | 483.65 (179.10) | 372.54 (180.43) | 324.00 (206.31) | 401.76 (171.55) |
| | **Acquiescence** | | | |
| | Senior opponent | Same-year opponent | Junior opponent | Total |
| 1st year (N=20) | 227.50 (251.15) | 72.22 (187.26)[a] | — | 189.38 (215.18) |
| 2nd year (N=16) | 187.50 (297.49) | 43.75 (296.58) | 62.50 (394.76) | 120.31 (256.62) |
| 3rd year (N=13) | 169.23 (209.70) | 7.69 (246.51) | 15.38 (316.48) | 51.92 (224.64) |
| 4th year (N=18) | — | -47.22 (269.24) | -101.85 (307.74) | -88.19 (287.05) |
| Total | 198.98 (253.46) | 19.23 (250.58) | -13.48 (342.41) | 71.64 (265.54) |

*Note.* SD is in parentheses. Some participants non-linearly responded when indicating which offers they would accept. Therefore, we could not calculate these participants' MAO and acquiescence, so they were excluded from the relevant analyses. Superscript 'a' indicates that two participants were excluded due to a non-linear response.

Table S3. The best model of acquiescence analyzed by GLMM containing pre-measured T as the fixed factors

| Effect | Estimates | SE | CI | degrees of freedom | T | p |
|---|---|---|---|---|---|---|
| **Intercept** | 6.848 | 0.042 | [6.765, 6.931] | 65 | 164.17 | <.0001 |
| **Opponent condition** | | | | | | |
| **Senior** | 0.187 | 0.041 | [0.105, 0.269] | 90 | 4.52 | <.0001 |
| **Same-year** | 0.046 | 0.037 | [-0.027, 0.118] | 90 | 1.25 | .213 |
| **Junior** | 0 | | | Contrast | | |
| **Pre-measured T** | -0.527 | 0.178 | [-0.883, -0.172] | 65 | -2.97 | .004 |
| **Opponent condition × T** | | | | | | |
| **Senior** | 0.420 | 0.179 | [0.065, 0.776] | 90 | 2.35 | .021 |
| **Same-year** | 0.285 | 0.159 | [-0.031, 0.601] | 90 | 1.79 | .077 |
| **Junior** | 0 | | | Contrast | | |
| **AIC** | 39.05 | | | | | |

*Note.* If the senior opponent condition was set to the contrast, it was revealed that the level of acquiescence in this condition was higher than in the other two conditions (same-year: $t = -3.88$, $p < .001$).

Table S4. The best model of acquiescence analyzed by GLMM containing pre-measured T and C as the fixed factors

| Effect | Estimates | SE | CI | degrees of freedom | T | p |
|---|---|---|---|---|---|---|
| **Intercept** | 6.855 | 0.041 | [6.772, 6.937] | 64 | 166.15 | <.0001 |
| **Opponent condition** | | | | | | |
| **Senior** | 0.181 | 0.041 | [0.099, 0.263] | 90 | 4.38 | <.0001 |
| **Same-year** | 0.043 | 0.037 | [-0.030, 0.115] | 90 | 1.17 | .247 |
| **Junior** | 0 | | | Contrast | | |
| **Pre-measured T** | -0.487 | 0.177 | [-0.841, -0.133] | 64 | -2.75 | .008 |
| **Pre-measured C** | -0.124 | 0.070 | [-0.264, 0.015] | 64 | -1.78 | .079 |
| **Opponent condition × T** | | | | | | |
| **Senior** | 0.415 | 0.178 | [0.060, 0.769] | 90 | 2.33 | .022 |
| **Same-year** | 0.275 | 0.159 | [-0.040, 0.591] | 90 | 1.73 | .087 |
| **Junior** | 0 | | | Contrast | | |
| **AIC** | 37.93 | | | | | |

*Note.* If the senior opponent condition was set to the contrast, it was revealed that the level of acquiescence in this condition was higher than in the other two conditions (same-year: $t = -3.81$, $p < .001$).

Table S5. Hormone levels in participants in both waves

| Testosterone | N | original value (pmol / L) | | | Log-transformed | |
|---|---|---|---|---|---|---|
| | | First | Second | Change | First | Second |
| 1st year in the first wave<br>3rd year in the second wave | 12 | 248.9<br>(62.3) | 264.7<br>(60.4) | 15.8<br>(75.2) | 5.49<br>(0.26) | 5.56<br>(0.22) |
| 2nd year in the first wave<br>4th year in the second wave | 18 | 281.5<br>(66.1) | 278.8<br>(76.6) | -2.7<br>(69.0) | 5.61<br>(0.24) | 5.60<br>(0.27) |
| Total | 30 | 268.4<br>(65.6) | 273.2<br>(69.8) | 4.7<br>(70.9) | 5.56<br>(0.25) | 5.58<br>(0.25) |

| Cortisol | N | original value (pmol / L) | | | Log-transformed | |
|---|---|---|---|---|---|---|
| | | First | Second | Change | First | Second |
| 1st year in the first wave<br>3rd year in the second wave | 12 | 2096.8<br>(817.4) | 6053.7<br>(2659.3) | 3956.9<br>(2787.9) | 7.57<br>(0.43) | 8.62<br>(0.45) |
| 2nd year in the first wave<br>4th year in the second wave | 18 | 4399.1<br>(2116.5) | 5803.1<br>(2687.3) | 1404.0<br>(2837.9) | 8.26<br>(0.56) | 8.56<br>(0.47) |
| Total | 30 | 3478.2<br>(2048.3) | 5903.3<br>(2632.8) | 2425.2<br>(3047.5) | 7.98<br>(0.61) | 8.59<br>(0.46) |

*Note.* The base of a logarithmic transformation is *e*, which is the base of the natural logarithm. SD is in parentheses.

Table S6. Behavior indexes in people who participated in both waves

| MAO | N | First | Second | Change |
|---|---|---|---|---|
| 1st year in the first wave<br>3rd year in the second wave | 12 | 236.8<br>(123.0) | 316.7<br>(167.3) | 79.9<br>(119.6) |
| 2nd year in the first wave<br>4th year in the second wave | 18 | 317.6[a]<br>(170.0) | 400.0[a]<br>(177.0) | 87.5[b]<br>(260.6) |
| Total | 30 | 284.2[a]<br>(155.2) | 365.5[a]<br>(175.1) | 84.2[b]<br>(208.7) |

| Offer | N | First | Second | Change |
|---|---|---|---|---|
| 1st year in the first wave<br>3rd year in the second wave | 12 | 408.3<br>(142.0) | 379.2<br>(147.6) | -29.2<br>(156.2) |
| 2nd year in the first wave<br>4th year in the second wave | 18 | 447.2<br>(156.0) | 297.9<br>(168.3) | -149.3<br>(229.6) |
| Total | 30 | 431.7<br>(149.3) | 330.4<br>(162.8) | -101.3<br>(209.2) |

| Acquiescence | N | First | Second | Change |
|---|---|---|---|---|
| 1st year in the first wave<br>3rd year in the second wave | 12 | 172.9<br>(114.0) | 62.5<br>(231.2) | -110.4<br>(182.3) |
| 2nd year in the first wave<br>4th year in the second wave | 18 | 126.5[a]<br>(203.2) | -93.4[a]<br>(295.0) | -213.3[b]<br>(357.6) |
| Total | 30 | 145.7[a]<br>(171.0) | -28.9[a]<br>(277.2) | -169.2[b]<br>(295.4) |

*Note.* SD is in parentheses. Some participants non-linearly responded when indicating which offers they would accept. Therefore, we could not calculate these participants' MAO and acquiescence, so they were excluded from the relevant analyses. Superscript 'a' indicates that one participant was excluded, and superscript 'b' indicates that two participants were excluded due to a non-linear response.

Table S7. The best model of acquiescence analyzed by GLMM containing pre-measured T as the fixed factors in the longitudinal analysis

| Effect | Estimates | SE | CI | degrees of freedom | T | p |
|---|---|---|---|---|---|---|
| **Intercept** | 7.113 | 0.063 | [6.984, 7.242] | 28 | 112.84 | <.0001 |
| **Wave** | | | | | | |
| **First** | 0 | | | Contrast | | |
| **Second** | -0.198 | 0.068 | [-0.338, -0.058] | 25 | -2.92 | .007 |
| **Seniority** | | | | | | |
| **First: 1st year Second: 3rd year** | 0 | | | Contrast | | |
| **First: 2nd year Second: 4th year** | -0.143 | 0.071 | [-0.287, 0.002] | 28 | -2.02 | .053 |
| **Pre-measured T** | 0.092 | 0.050 | [-0.012, 0.195] | 25 | 1.83 | .080 |
| **Wave × T** | | | | | | |
| **First** | 0 | | | Contrast | | |
| **Second** | -0.209 | 0.071 | [-0.355, -0.063] | 25 | -2.95 | .007 |
| **AIC** | 19.31 | | | | | |

*Note.* Pre-measured T was standardized by each experiment.

Table S8. The best model of acquiescence analyzed by GLMM containing pre-measured T and C as the fixed factors in the longitudinal analysis

| Effect | Estimates | SE | CI | degrees of freedom | T | p |
|---|---|---|---|---|---|---|
| **Intercept** | 7.098 | 0.064 | [6.967, 7.229] | 28 | 110.98 | <.0001 |
| **Wave** | | | | | | |
| **First** | 0 | | Contrast | | | |
| **Second** | -0.160 | 0.068 | [-0.302, -0.018] | 21 | -2.34 | .029 |
| **Seniority** | | | | | | |
| **First: 1st year Second: 3rd year** | 0 | | Contrast | | | |
| **First: 2nd year Second: 4th year** | -0.126 | 0.072 | [-0.272, 0.021] | 28 | -1.75 | .090 |
| **Pre-measured T** | 0.105 | 0.053 | [-0.005, 0.215] | 21 | 1.98 | .061 |
| **Pre-measured C** | -0.037 | 0.054 | [-0.149, 0.074] | 21 | -0.69 | .496 |
| **Wave × T** | | | | | | |
| **First** | 0 | | Contrast | | | |
| **Second** | -0.190 | 0.072 | [-0.341, -0.040] | 21 | -2.63 | .016 |

| Effect | | Estimates | SE | CI | degrees of freedom | T | p |
|---|---|---|---|---|---|---|---|
| **Wave × C** | | | | | | | |
| | **First** | 0 | | | Contrast | | |
| | **Second** | -0.034 | 0.072 | [-0.184, 0.116] | 21 | -0.47 | .641 |
| **T × C** | | 0.018 | 0.071 | [-0.129, 0.165] | 21 | 0.25 | .804 |
| **Wave × T × C** | | | | | | | |
| | **First** | 0 | | | Contrast | | |
| | **Second** | -0.151 | 0.088 | [-0.335, 0.033] | 21 | -1.71 | .102 |
| **AIC** | | 19.05 | | | | | |

*Note.* Pre-measured T and C were standardized by each experiment.

## Supplementary Information 2. Analyses of MAO and Offer

We analyzed MAO and offer by the same method as acquiescence. Because the lognormal distribution cannot be defined when the value is 0, we added 100, the minimum increment of decision, to both MAO and offer for GLMMs.

First, we conducted GLMM twice for each behavioral index of the second wave. In the first analysis, we set seniority, opponent conditions, pre-measured T, and each interaction as the fixed effects, and participants as the random effect. We then conducted GLMM, adding pre-measured C and relevant interactions as fixed effects. T and C were centered in order to facilitate the interpretation of estimated coefficients.

In the GLMM analysis of MAO without C, a model containing the main effect of the opponent condition and T was selected as the best model (Table S9). MAO in the senior opponent condition was significantly lower than in the other two conditions. Furthermore, unlike in Inoue et al.[1], T had a significantly positive effect on MAO: participants who had a higher baseline T claimed more money. These main effects were also significant when C and relevant interactions were added as a fixed effect (Table S10). In the GLMM analysis of MAO with C, the model adding the main effects of seniority, C, and interaction between these was selected as the best model. In addition to the main effects of opponent condition and T, the interaction between seniority and C was significant. This interaction indicated a negative correlation between C and MAO among the first-year students, although there were positive correlations among the other three years (Figure S1).

Table S9. The best model of MAO analyzed by GLMM containing pre-measured T as fixed factors

| Effect | Estimates | SE | CI | degrees of freedom | t | p |
|---|---|---|---|---|---|---|
| **Intercept** | 5.817 | 0.066 | [5.686, 5.948] | 65 | 88.78 | <.0001 |
| **Opponent condition** | | | | | | |
| **Senior** | 0 | | | Contrast | | |
| **Same-year** | 0.198 | 0.061 | [0.077, 0.318] | 92 | 3.26 | .002 |
| **Junior** | 0.155 | 0.068 | [0.019, 0.291] | 92 | 2.27 | .026 |
| **Pre-measured T** | 0.555 | 0.211 | [0.134, 0.976] | 65 | 2.63 | .011 |
| **AIC** | 191.42 | | | | | |

Table S10. The best model of MAO analyzed by GLMM containing pre-measured T and C as fixed factors

| Effect | Estimates | SE | CI | degrees of freedom | t | p |
|---|---|---|---|---|---|---|
| **Intercept** | 5.784 | 0.110 | [5.563, 6.004] | 58 | 52.42 | <.0001 |
| **Opponent condition** | | | | | | |
| **Senior** | 0 | | | Contrast | | |
| **Same-year** | 0.194 | 0.062 | [0.072, 0.316] | 92 | 3.15 | .002 |
| **Junior** | 0.150 | 0.071 | [0.010, 0.291]] | 92 | 2.12 | .036 |
| **Seniority** | | | | | | |
| **1st year** | 0 | | | Contrast | | |
| **2nd year** | -0.238 | 0.151 | [-0.540, 0.064] | 58 | -1.58 | .120 |
| **3rd year** | -0.021 | 0.158 | [-0.338, 0.296] | 58 | -0.13 | .896 |
| **4th year** | 0.087 | 0.145 | [-0.204, 0.378] | 58 | 0.60 | .551 |
| **Pre-measured T** | 0.444 | 0.193 | [0.057, 0.831] | 58 | 2.30 | .025 |
| **Pre-measured C** | -0.211 | 0.195 | [-0.601, 0.179] | 58 | -1.08 | .283 |
| **Seniority × C** | | | | | | |
| **1st year** | 0 | | | Contrast | | |
| **2nd year** | 0.973 | 0.339 | [0.294, 1.653] | 58 | 2.87 | .006 |
| **3rd year** | 0.535 | 0.311 | [-0.087, 1.158] | 58 | 1.72 | .091 |
| **4th year** | 0.642 | 0.269 | [0.105, 1.180] | 58 | 2.39 | .020 |
| **AIC** | 188.95 | | | | | |

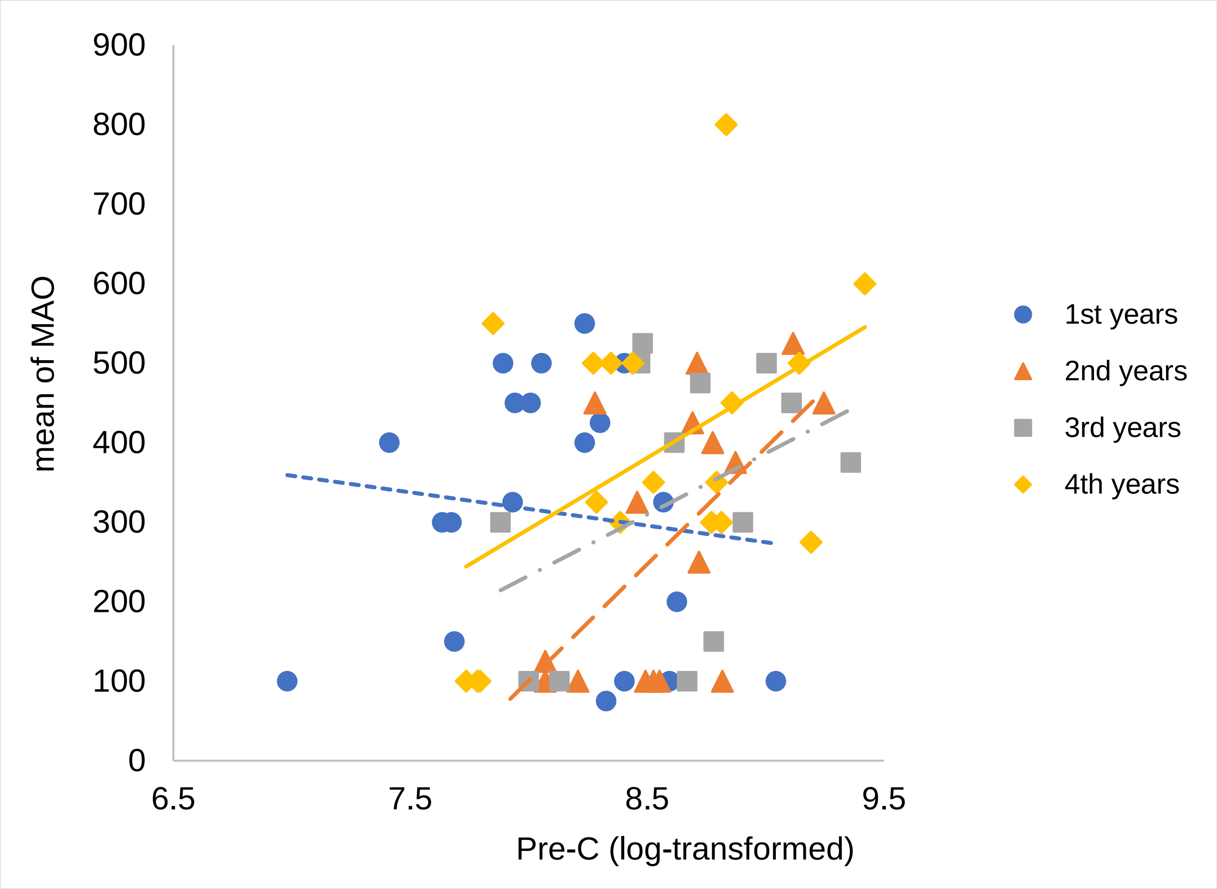


Figure S1. The relationship between baseline C level and the mean of MAO for each seniority level.

*Note.* Spearman's correlation coefficient: first years ($r_s$ = -.17, $p$ = .46), second years ($r_s$ = .49, $p$ = .054), third years ($r_s$ = .27, $p$ = .38), fourth years ($r_s$ = .40, $p$ = .10).

On the contrary, the best model for offer did not contain T, unlike in the first wave[1]. Both analyses (without and with C) selected the same model as the best model (Table S11). The best model contained significant main effects of opponent condition and seniority. The main effect of opponent condition indicated that participants offered more in the senior opponent condition than in the other two conditions. The main effect of seniority indicated that the first-year students offered significantly more than second-year and fourth-year students.

Table S11. The best model of the offer analyzed by GLMM containing pre-measured T and C as fixed factors

| Effect | Estimates | SE | CI | degrees of freedom | t | p |
|---|---|---|---|---|---|---|
| **Intercept** | 6.460 | 0.095 | [6.271, 6.649] | 67 | 68.19 | <.0001 |
| **Opponent condition** | | | | | | |
| **Senior** | 0 | | Contrast | | | |
| **Same-year** | -0.186 | 0.064 | [-0.370, -0.076] | 100 | -2.91 | .004 |
| **Junior** | -0.223 | 0.074 | [-0.312, -0.059] | 100 | -3.02 | .003 |
| **Seniority** | | | | | | |
| **1st year** | 0 | | Contrast | | | |
| **2nd year** | -0.295 | 0.129 | [-0.552, -0.037] | 67 | -2.28 | .026 |
| **3rd year** | -0.210 | 0.141 | [-0.491, 0.071] | 67 | -1.49 | .140 |
| **4th year** | -0.411 | 0.135 | [-0.679, -0.142] | 67 | -3.05 | .003 |
| **AIC** | 214.76 | | | | | |

Next, we conducted the longitudinal analysis of MAO and offer using the same method as for acquiescence. In the first GLMM, we put the waves (first/second), the seniority, pre-measured T, and each interaction as the fixed effect. The random effect was participants. After that, we conducted the second GLMM, adding pre-measured C and relevant interactions as fixed effects. T and C were standardized for each wave. In the first analysis of MAO, the model containing only the main effect of the wave was the best model (Table S12). This significant main effect indicates that participants claimed more in the second wave (when they were more senior) than in the first wave. However, after adding C as a fixed effect, the main effect of the wave was not significant. When C and relevant interactions were added as the fixed effect, the best model contains all main effects, the interaction between the wave and T, and the interaction between the seniority and T. The main effect of C and the interaction between seniority and T were significant (Table S13). Participants with higher baseline C claimed more than participants with lower C. In addition, the interaction meant that the correlation coefficients between T and MAO were higher in the lower years (first years in the first wave and third years in the second wave) than in the higher years (second years in the first wave and fourth years in the second wave) in both waves (Figure S2). We must note that the covariance parameter estimate of the random effect was zero in this model with C. As with the analyses presented in the main text, the random effects were left in the model based on the experimental design, but the model estimations were the same as those without the random effects (cf.[2]).

Regarding the offer, the same model was selected as the best model by both GLMMs. This model contains the main effects of wave, seniority, T and the interaction between seniority and T (Table S14). Only the main effect of T was significant. This means that those who has higher baseline T offered more. In this model, the covariance parameter estimate of the random effect was also zero.

Table S12. The best model of MAO analyzed by GLMM containing pre-measured T as one of the fixed factors in the longitudinal analysis

| Effect | | Estimates | SE | CI | degrees of freedom | t | p |
|---|---|---|---|---|---|---|---|
| **Intercept** | | 5.841 | 0.089 | [5.659, 6.023] | 29 | 65.65 | <.0001 |
| **Wave** | | | | | | | |
| | **First** | 0 | | | Contrast | | |
| | **Second** | 0.227 | 0.109 | [0.003, 0.451] | 27 | 2.08 | .048 |
| | **AIC** | 85.46 | | | | | |

Table S13. The best model of MAO analyzed by GLMM containing pre-measured T and C as the fixed factors in the longitudinal analysis

| Effect | | Estimates | SE | CI | degrees of freedom | t | p |
|---|---|---|---|---|---|---|---|
| **Intercept** | | 5.809 | 0.107 | [5.589, 6.028] | 28 | 54.13 | <.0001 |
| **Wave** | | | | | | | |
| | **First** | 0 | | Contrast | | | |
| | **Second** | 0.194 | 0.113 | [-0.039, 0.428] | 23 | 1.72 | .098 |
| **Seniority** | | | | | | | |
| | **First: 1st year Second: 3rd year** | 0 | | Contrast | | | |
| | **First: 2nd year Second: 4th year** | 0.123 | 0.120 | [-0.122, 0.368] | 28 | 1.03 | .313 |
| **Pre-T** | | 0.060 | 0.106 | [-0.159, 0.279] | 23 | 0.57 | .577 |
| **Pre-C** | | 0.137 | 0.061 | [0.012, 0.262] | 23 | 2.27 | .033 |
| **Wave × Pre-T** | | | | | | | |
| | **First** | 0 | | Contrast | | | |
| | **Second** | 0.222 | 0.118 | [-0.022, 0.467] | 23 | 1.88 | .073 |
| **Seniority × Pre-T** | | | | | | | |
| | **First: 1st year Second: 3rd year** | 0 | | Contrast | | | |
| | **First: 2nd year Second: 4th year** | -0.290 | 0.124 | [-0.546, -0.034] | 23 | -2.34 | .028 |
| | **AIC** | 81.81 | | | | | |

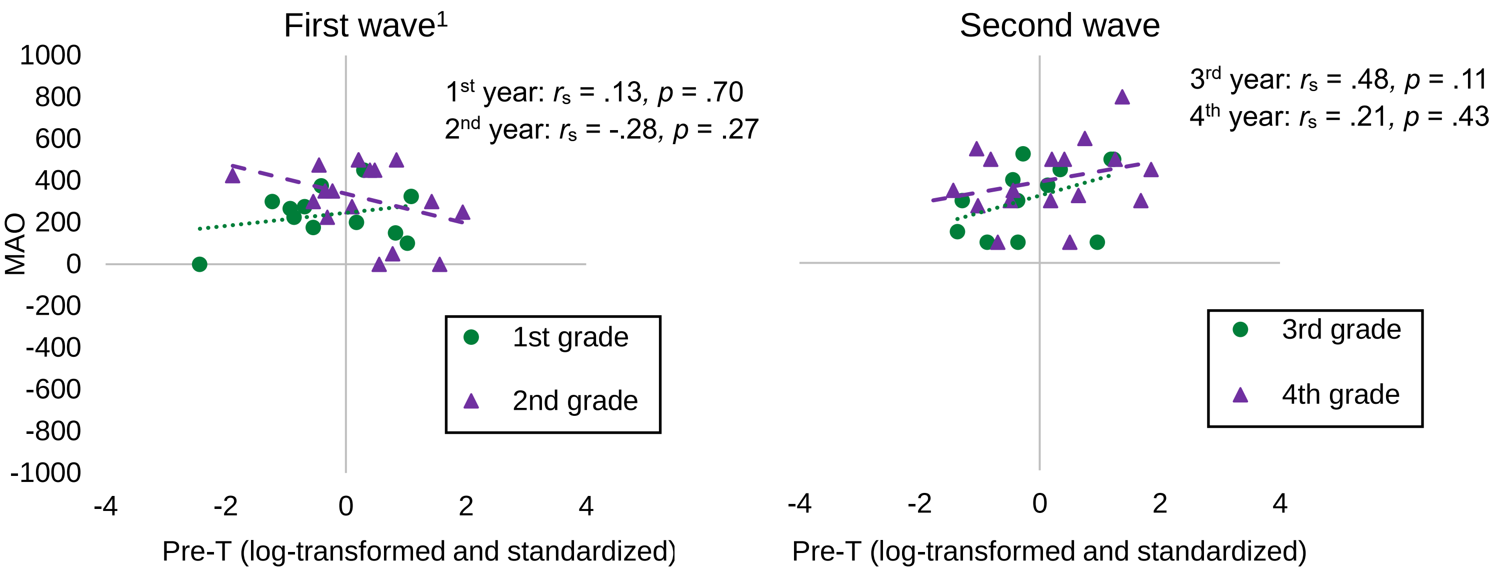


Figure S2.

The relationship between pre-game T level and MAO in each year

Table S14. The best model of the offer analyzed by GLMM containing pre-measured T and C as fixed factors in the longitudinal analysis

| Effect | Estimates | SE | CI | degrees of freedom | t | p |
|---|---|---|---|---|---|---|
| **Intercept** | 6.284 | 0.108 | [6.062, 6.506] | 28 | 57.99 | <.0001 |
| **Wave** | | | | | | |
| **Previous** | 0 | | Contrast | | | |
| **Current** | -0.229 | 0.115 | [-0.465, 0.007] | 27 | -1.99 | .057 |
| **Seniority** | | | | | | |
| **First: 1st year<br>Second: 3rd year** | 0 | | Contrast | | | |
| **First: 2nd year<br>Second: 4th year** | -0.137 | 0.119 | [-0.381, 0.108] | 28 | -1.14 | .263 |
| **Pre-T** | 0.199 | 0.084 | [0.027, 0.372] | 27 | 2.37 | .025 |
| **Seniority × Pre-T** | | | | | | |
| **First: 1st year<br>Second: 3rd year** | 0 | | Contrast | | | |
| **First: 2nd year<br>Second: 4th year** | -0.215 | 0.117 | [-0.455, 0.026] | 27 | -1.83 | .079 |
| **AIC** | 85.18 | | | | | |

## Supplementary Information 3. Analyses using hormones before transformation

In the main text, we reported analyses using log-transformed hormones because they were not normally distributed. For robustness confirmation, here we reported analyses of the data of the second wave using baseline hormones before transformation.

The results were almost the same as the analyses using log-transformed hormones. The only difference is the interaction between Opponent condition and T became significant. This interaction was significant between junior opponent condition and same-year opponent condition, which is marginally significant in analyses using log-transformed hormones.

Table S15. The best model of acquiescence analyzed by GLMM containing pre-measured T before transformation as the fixed factors

| Effect | Estimates | SE | CI | degrees of freedom | T | p |
|---|---|---|---|---|---|---|
| **Intercept** | 6.844 | 0.042 | [6.761, 6.928] | 65 | 164.12 | <.0001 |
| **Opponent condition** | | | | | | |
| **Senior** | 0.191 | 0.041 | [0.109, 0.273] | 90 | 4.62 | <.0001 |
| **Same-year** | 0.049 | 0.037 | [-0.024, 0.121] | 90 | 1.33 | .186 |
| **Junior** | 0 | | Contrast | | | |
| **Pre-measured T** | -0.0019 | 0.0006 | [-0.0032, -0.0007] | 65 | -3.08 | .003 |
| **Opponent condition × T** | | | | | | |
| **Senior** | 0.0016 | 0.0006 | [0.0003, 0.0028] | 90 | 2.46 | .016 |
| **Same-year** | 0.0012 | 0.0006 | [0.0001, 0.0023] | 90 | 2.12 | .037 |
| **Junior** | 0 | | Contrast | | | |
| **AIC** | 38.57 | | | | | |

*Note.* If the senior opponent condition was set to the contrast, it was revealed that the level of acquiescence in this condition was higher than in the other two conditions (same-year: $t = -3.92$, $p < .001$).

Table S16. The best model of acquiescence analyzed by GLMM containing pre-measured T and C before transformation as the fixed factors

| Effect | Estimates | SE | CI | degrees of freedom | T | p |
|---|---|---|---|---|---|---|
| **Intercept** | 6.851 | 0.041 | [6.769, 6.933] | 64 | 166.44 | <.0001 |
| **Opponent condition** | | | | | | |
| **Senior** | 0.185 | 0.041 | [0.103, 0.268] | 90 | 4.49 | <.0001 |
| **Same-year** | 0.045 | 0.037 | [-0.027, 0.118] | 90 | 1.24 | .219 |
| **Junior** | 0 | | Contrast | | | |
| **Pre-measured T** | -0.0018 | 0.0006 | [-0.0030, -0.0006] | 64 | -2.90 | .005 |
| **Pre-measured C** | -0.00003 | 0.00001 | [-0.0001, 0.0000] | 64 | -1.87 | .067 |
| **Opponent condition × T** | | | | | | |
| **Senior** | 0.0015 | 0.0006 | [0.0002, 0.0027] | 90 | 2.37 | .020 |
| **Same-year** | 0.0011 | 0.0006 | [0.0000, 0.0023] | 90 | 2.03 | .045 |
| **Junior** | 0 | | Contrast | | | |
| **AIC** | 37.16 | | | | | |

*Note.* If the senior opponent condition was set to the contrast, it was revealed that the level of acquiescence in this condition was higher than in the other two conditions (same-year: $t = -3.86$, $p < .001$).

## Supplementary Information 4. The analyses of post-experiment hormones

Here, we report the association between post-experiment T levels and behavior in the UG. Note that participants' post-experiment saliva samples were collected after intense physical exercise (regular practice). Since T and C levels rise in response to physical exercise[3], the post-experiment hormone levels were influenced by this exercise.

We analyzed post-experiment hormones (T and C) before transformation by the GLMM with fixed effects of participants' seniority and the average behavioral indexes (acquiescence, MAO, offer) across all opponent conditions. No random effect was applied as there was no within-participant factor. We adopted the lognormal distribution in all GLMMs with the identity link function. The parameters were estimated using the Laplace approximation.

There were no significant effects of behavioral indexes to post-experiment hormones levels. By contrast, a significant effect of seniority to post-experiment C was found by GLMMs where acquiscence and offer were initially included as fixed effects. Specifically, post-experiment C levels were significantly lower for first-year students than for second- and fourth-year students.

Table S17. The best model of post-experiment T analyzed by GLMM containing average acquiescence and seniority as the fixed factors

| Effect | Estimates | SE | CI | degrees of freedom | T | p |
|---|---|---|---|---|---|---|
| **Intercept** | 5.224 | 0.059 | [5.107, 5.341] | 65 | 89.16 | <.0001 |
| **Mean acquiescence** | $3.716 \times 10^{-6}$ | 0.0002 | [-0.0004, 0.0004] | 65 | 0.02 | .986 |
| **AIC** | 92.89 | | | | | |

Table S18. The best model of post-experiment C analyzed by GLMM containing average acquiescence and seniority as the fixed factors

| Effect | Estimates | SE | CI | degrees of freedom | T | p |
|---|---|---|---|---|---|---|
| **Intercept** | 7.729 | 0.126 | [7.477, 7.982] | 62 | 61.13 | <.0001 |
| **Mean acquiescence** | 0.0005 | 0.0003 | [-0.00004, 0.0010] | 62 | 1.86 | .068 |
| **Seniority** | | | | | | |
| **1st year** | 0 | | contrast | | | |
| **2nd year** | 0.417 | 0.175 | [0.067, 0.767] | 62 | 2.38 | .020 |
| **3rd year** | 0.254 | 0.189 | [-0.123, 0.630] | 62 | 1.34 | .184 |
| **4th year** | 0.462 | 0.184 | [0.095, 0.830] | 62 | 2.51 | .015 |
| **AIC** | 114.35 | | | | | |

Table S19. The best model of post-experiment T analyzed by GLMM containing average MAO and seniority as the fixed factors

| Effect | Estimates | SE | CI | degrees of freedom | T | p |
|---|---|---|---|---|---|---|
| **Intercept** | 5.170 | 0.122 | [4.927, 5.413] | 65 | 42.51 | <.0001 |
| **Mean MAO** | 0.0002 | 0.0003 | [-0.0005, 0.0008] | 65 | 0.51 | .615 |
| **AIC** | 92.64 | | | | | |

Table S20. The best model of post-experiment C analyzed by GLMM containing average MAO and seniority as the fixed factors

| Effect | Estimates | SE | CI | degrees of freedom | T | p |
|---|---|---|---|---|---|---|
| **Intercept** | 8.114 | 0.146 | [7.823, 8.405] | 65 | 55.71 | <.0001 |
| **Mean MAO** | -0.0002 | 0.0004 | [-0.0010, 0.0006] | 65 | -0.60 | .553 |
| **AIC** | 116.81 | | | | | |

Table S21. The best model of post-experiment T analyzed by GLMM containing average offer and seniority as the fixed factors

| Effect | Estimates | SE | CI | degrees of freedom | T | p |
|---|---|---|---|---|---|---|
| **Intercept** | 5.229 | 0.138 | [4.954, 5.504] | 69 | 37.92 | <.0001 |
| **Offer** | $5.855 \times 10^{-6}$ | 0.0003 | [-0.0006, 0.0006] | 69 | 0.02 | .985 |
| **AIC** | 95.20 | | | | | |

Table S22. The best model of post-experiment C analyzed by GLMM containing average offer and seniority as the fixed factors

| Effect | Estimates | SE | CI | degrees of freedom | T | p |
|---|---|---|---|---|---|---|
| **Intercept** | 7.801 | 0.118 | [7.565, 8.037] | 67 | 61.13 | <.0001 |
| **Seniority** | | | | | | |
| **1st year** | 0 | | Contrast | | | |
| **2nd year** | 0.351 | 0.174 | [0.004, 0.698] | 67 | 2.02 | .047 |
| **3rd year** | 0.207 | 0.191 | [-0.174, 0.588] | 67 | 1.08 | .282 |
| **4th year** | 0.405 | 0.171 | [0.063, 0.747] | 67 | 2.36 | .021 |
| **AIC** | 124.28 | | | | | |

## Supplementary Information 5.
## Reanalysis of the data of the first wave (Inoue et al., 2017)

Since we did not conduct analyses considering C in the first wave[1] , we reanalyzed the data by adding C as one of the independent variables. For these analyses, we used GLMM instead of the general linear model we did in the previous paper[1]. This is because the dependent variables (behavioral indexes)[i] were non-normally distributed.

We analyzed acquiescence, MAO, and offer using GLMM. In these GLMMs, we set seniority, the game type (details were described below), pre-measured T, pre-measured C, and each interaction as the fixed effects. The random effect was participants. Prior to these analyses, T and C were centered in order to make the interpretation of estimated coefficients easier. We adopted the lognormal distribution in all GLMMs. To prepare for these analyses, we transformed behavioral indexes to positive values: we added 1000 to acquiescence and 100 to MAO and offer.

We used the glimmix procedure of SAS in these analyses. We adopted the lognormal distribution in all GLMMs and set the link function as identity. The parameters were estimated using the laplace approximation. We selected variables by backward selection and chose the best model based on Akaike's Information Criterion (AIC).

The baseline C data was obtained from ASKA Pharma Medical Co., to which we outsourced the saliva assay in LC-MS/MS. For details of the experimental procedure of the first wave, please see the previous paper[1]. Note that the game types of the first wave are different from the second wave, as mentioned in the main text. In the first wave, participants played each of four different games once[1]; "no information game," "peer-to-peer game",

---

[i] We reanalyzed the data reported in the first wave using GLMM and found no differences in the main results.

“fourth-year game”, and “first-year game.” In the “no information game,” participants played without any information about their opponents’ seniority. In the “peer-to-peer game,” participants played against opponents from the same year. In the “fourth-year game”, fourth-year students played against junior opponents (first to third years). In the “first-year game”, first-year students played against senior opponents (second to fourth years). These four game types were set as the fixed variable in GLMMs.

The descriptive statistics of pre-game C (and T) are shown in Table S23. There was a significant difference in pre-game C as a function of participants’ seniority ($F$ (3, 66) = 5.99, $p < .01$, $\eta_p^2 = .214$). According to the multiple comparisons, the pre-game C of the first year was significantly lower than that of the second and fourth years. Pre-game C and T were positively correlated ($r_s = .25$, $p = .04$). Most previous studies have reported positive correlations (e.g.,[4–7]) or non-significant correlations (e.g.,[6,8]) between T and C, which was consistent with our result.

Table S23. The value of pre-measured hormones in the first wave

| | N | original value (pmol / L) | | Log-transformed | |
|---|---|---|---|---|---|
| | | T | C | T | C |
| 1st year | 12 | 248.9 (62.3) | 2096.8 (817.4) | 5.49 (0.26) | 7.57 (0.43) |
| 2nd year | 22 | 274.4 (63.6) | 4370.5 (2206.9) | 5.59 (0.23) | 8.23 (0.60) |
| 3rd year | 14 | 304.0 (114.9) | 3902.0 (2693.7) | 5.66 (0.34) | 7.95 (0.93) |
| 4th year | 22 | 279.4 (61.4) | 5495.4 (2865.9) | 5.61 (0.23) | 8.49 (0.52) |
| All | 70 | 277.5 (76.0) | 4240.6 (2604.9) | 5.59 (0.26) | 8.14 (0.70) |

*Note.* The base of a logarithmic transformation is *e*, which is the base of the natural logarithm. SD is in parenthesis.

First, we conducted GLMM for acquiescence. We set seniority, game type, pre-measured T, pre-measured C, and each interaction as fixed effects. The best model is shown in Table S24.

In this model, the main effects of game type, seniority, T, and the interaction between seniority and T were significant when the fourth-year game and the fourth year were set as a contrast. The level of acquiescence in the fourth-year game were greater than in the other three game types. The differences between the fourth-year game and the no-information game and the first-year game were significant. In addition, fourth year players acquiesced significantly less than those in the first and second years. Regarding T, a higher baseline T was associated with a lower level of acquiescence in the fourth year. However, more importantly, the interaction between seniority and T was significant. This result suggests that the relationship between higher T and lower acquiescence was observed only in the fourth years. Conversely, in the other three years, participants who had higher T tended to acquiescence more. These results were almost identical to the GLM without C in the previous paper[1]. As a C-relevant effect, the interaction between C and seniority was marginally significant between the second and fourth When the second grade was set as the contrast, there was a significant difference in this interaction between the third grades (see the note in Table S24). This was because the negative correlation between C and acquiescence was strongest in the second years (Figure S3).

Table S24. The best model of acquiescence analyzed by GLMM containing pre-measured T and C as the fixed factors in the first wave[1]

| Effect | Estimates | SE | CI | degrees of freedom | t | p |
|---|---|---|---|---|---|---|
| **Intercept** | 6.966 | 0.035 | [6.897, 7.036] | 55 | 200.76 | <.0001 |
| **Game type** | | | | | | |
| **No information** | -0.053 | 0.026 | [-0.105, -0.001] | 191 | -2.00 | .047 |
| **Peer to peer** | -0.046 | 0.026 | [-0.098, 0.006] | 191 | -1.76 | .081 |
| **First year** | -0.080 | 0.026 | [-0.132, -0.028] | 191 | -3.01 | .003 |
| **Fourth year** | 0 | | | Contrast | | |
| **Seniority** | | | | | | |
| **1st year** | 0.149 | 0.066 | [0.016, 0.282] | 55 | 2.25 | .028 |
| **2nd year** | 0.102 | 0.040 | [0.021, 0.183] | 55 | 2.53 | .014 |
| **3rd year** | 0.053 | 0.045 | [-0.037, 0.144] | 55 | 1.18 | .241 |
| **4th year** | 0 | | | Contrast | | |
| **Pre-measured T** | -0.261 | 0.115 | [-0.491, -0.031] | 55 | -2.27 | .027 |
| **Pre-measured C** | -0.009 | 0.050 | [-0.109, 0.091] | 55 | -0.18 | .862 |
| **Seniority × T** | | | | | | |
| **1st year** | 0.462 | 0.177 | [0.108, 0.816] | 55 | 2.62 | .012 |
| **2nd year** | 0.774 | 0.170 | [0.433, 1.114] | 55 | 4.56 | <.0001 |
| **3rd year** | 0.433 | 0.166 | [0.101, 0.765] | 55 | 2.62 | .011 |
| **4th year** | 0 | | | Contrast | | |
| **Seniority × C** | | | | | | |
| **1st year** | -0.001 | 0.095 | [-0.192, 0.188] | 55 | -0.02 | .986 |
| **2nd year** | -0.131 | 0.068 | [-0.267, 0.004] | 55 | -1.94 | .057 |
| **3rd year** | 0.046 | 0.061 | [-0.076, 0.168] | 55 | 0.76 | .452 |

| Effect | Estimates | SE | CI | degrees of freedom | t | p |
|---|---|---|---|---|---|---|
| **4th year** | 0 | | | Contrast | | |
| **AIC** | -157.28 | | | | | |

*Note.* If the 2nd year was set to the contrast at the seniority, it was revealed that there was a significant difference between 2nd and 3rd years in the interaction between the seniority and C ($t$ = -3.09, $p$ = .003).

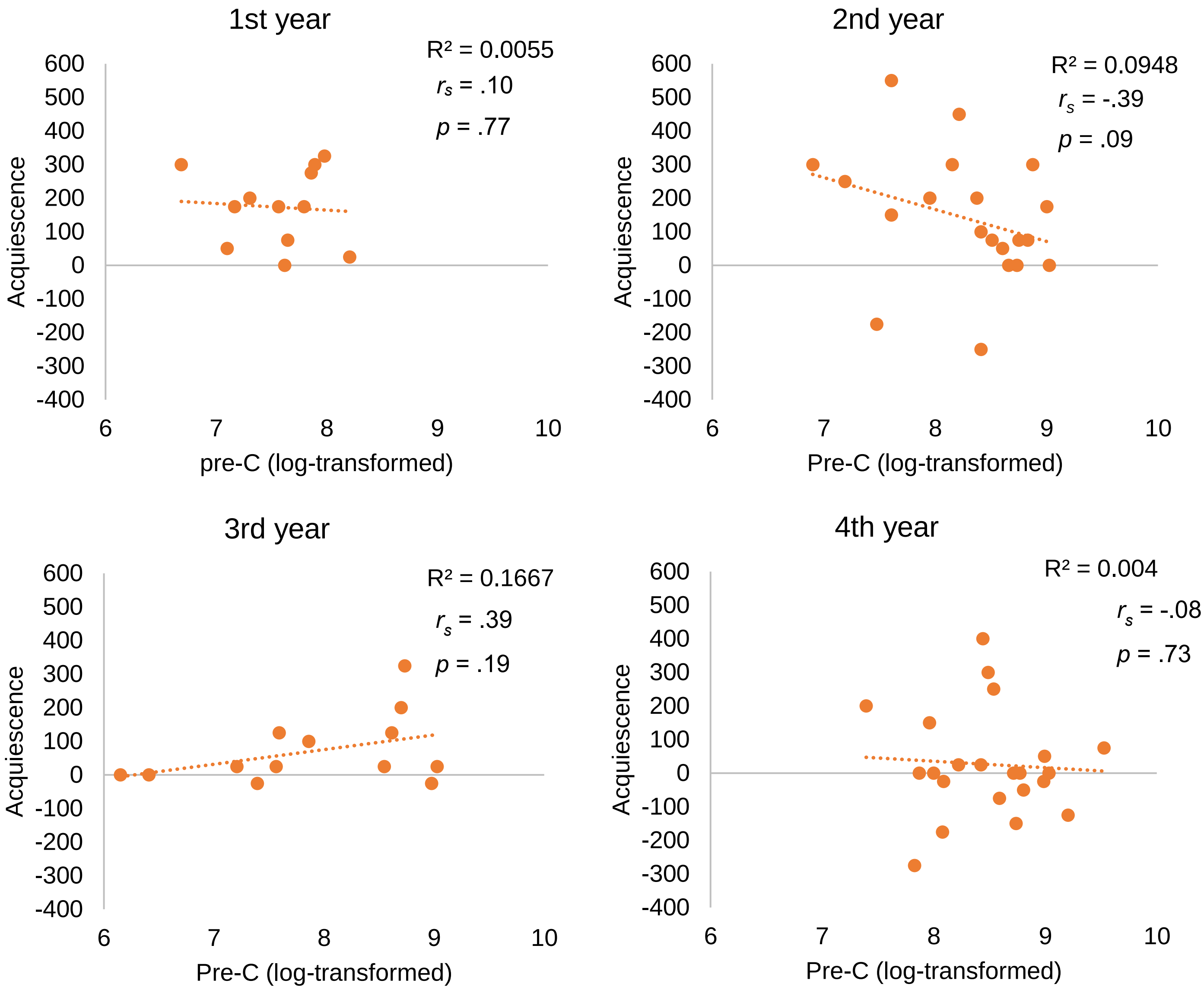


Figure S3. The relationship between pre-game C level and acquiescence by year in the first wave[1]

Next, we reanalyzed the other two behavioral indexes, MAO and offer, using GLMM. We conducted GLMM twice for each index. Fixed effects were seniority, game type, pre-measured T, pre-measured C, and each interaction. The random effect was participants. Before analyses, T and C were centered in order to facilitate the interpretation of estimated coefficients.

In the analysis of MAO, the model containing only the main effect of game type was selected (Table S25). MAO in the fourth-year game was lower than that in the other three game types, and the difference in MAO between the fourth-year game and the no information game was significant. The effect of T was excluded in the best model.

On the other hand, the best model of offer contained the main effects of seniority, T, C, and the interaction between T and C (Table S26). The main effect of seniority indicated that fourth-year students offered significantly less than first and second-year students. The main effects of T revealed that those with higher baseline T offered significantly more. In addition, the significant interaction was partially consistent with the dual-hormone hypothesis: higher C weakened the effect of T.

Table S25. The best model of MAO analyzed by GLMM containing pre-measured T and C as the fixed factors in the first wave[1]

| Effect | Estimates | SE | CI | degrees of freedom | t | p |
|---|---|---|---|---|---|---|
| **Intercept** | 5.699 | 0.077 | [5.544, 5.853] | 66 | 73.70 | <.0001 |
| **Game type** | | | | | | |
| **No information** | 0.156 | 0.056 | [0.046, 0.266] | 191 | 2.80 | .006 |
| **Peer to peer** | 0.107 | 0.056 | [-0.003, 0.217] | 191 | 1.92 | .056 |
| **First year** | 0.102 | 0.056 | [-0.008, 0.212] | 191 | 1.82 | .070 |
| **Fourth year** | 0 | Contrast | | | | |
| **AIC** | 321.38 | | | | | |

Table S26. The best model of offer analyzed by GLMM containing pre-measured T and C as the fixed factors in the first wave[1]

| Effect | Estimates | SE | CI | degrees of freedom | t | p |
|---|---|---|---|---|---|---|
| **Intercept** | 5.852 | 0.103 | [5.647, 6.057] | 63 | 57.08 | <.0001 |
| **Seniority** | | | | | | |
| **1st year** | 0.405 | 0.183 | [0.039, 0.771] | 63 | 2.21 | .031 |
| **2nd year** | 0.398 | 0.140 | [0.119, 0.677] | 63 | 2.85 | .006 |
| **3rd year** | 0.194 | 0.164 | [-0.135, 0.522] | 63 | 1.18 | .243 |
| **4th year** | 0 | | | Contrast | | |
| **Pre-measured T** | 0.545 | 0.219 | [0.106, 0.983] | 63 | 2.48 | .016 |
| **Pre-measured C** | 0.013 | 0.090 | [-0.167, 0.194] | 63 | 0.15 | .883 |
| **Pre-T × Pre-C** | -0.902 | 0.383 | [-1.666, -0.138] | 63 | -2.36 | .022 |
| **AIC** | 299.07 | | | | | |

## References


1. Inoue, Y. *et al.* Testosterone promotes either dominance or submissiveness in the Ultimatum Game depending on players' social rank. *Sci. Rep.* **7**, 5335 (2017).
2. Kiernan, K., Tao, J. & Gibbs, P. Tips and strategies for mixed modeling with SAS/STAT® procedures. *SASSTAT® Softw. Pap.* **332**, 18 (2012).
3. Brownlee, K. K., Moore, A. W. & Hackney, A. C. Relationship Between Circulating Cortisol and Testosterone: Influence of Physical Exercise. *J. Sports Sci. Med.* **4**, 76–83 (2005).
4. Mehta, P. H. & Josephs, R. A. Testosterone and cortisol jointly regulate dominance: Evidence for a dual-hormone hypothesis. *Horm. Behav.* **58**, 898–906 (2010).
5. Popma, A. *et al.* Cortisol moderates the relationship between testosterone and aggression in delinquent male adolescents. *Biol. Psychiatry* **61**, 405–411 (2007).
6. Edwards, D. A. & Casto, K. V. Women's intercollegiate athletic competition: Cortisol, testosterone, and the dual-hormone hypothesis as it relates to status among teammates. *Horm. Behav.* **64**, 153–160 (2013).
7. Pfattheicher, S. Illuminating the dual-hormone hypothesis: About chronic dominance and the interaction of cortisol and testosterone. *Aggress. Behav.* **43**, 85–92 (2017).
8. Pfattheicher, S., Landhäußer, A. & Keller, J. Individual differences in antisocial punishment in public goods situations: The interplay of cortisol with testosterone and dominance. *J. Behav. Decis. Mak.* **27**, 340–348 (2014).

## Supplementary Information 6. Experimental materials

There follow translations of participant instruction sheets (1), and decision sheets for each condition and tailored for each level of participant seniority (2 – 5).

1: Instructions for all participants.

2: Decision sheet in the first decision (vs. same year) for all participants.

3: Decision sheet in the second decision (1st year vs. 4th year / 2nd year vs. 3rd year).

- 3-1: For the 1st year students.
- 3-2: For the 2nd year students.
- 3-3: For the 3rd year students.
- 3-4: For the 4th year students.

4: Decision sheet in the third decision (1st year vs. 3rd year / 2nd year vs. 4th year).

- 4-1: For the 1st year students.
- 4-2: For the 2nd year students.
- 4-3: For the 3rd year students.
- 4-4: For the 4th year students.

5: Decision sheet in the fourth decision (1st year vs. 2nd year / 3rd year vs. 4th year).

- 5-1: For the 1st year students.
- 5-2: For the 2nd year students.
- 5-3: For the 3rd year students.
- 5-4: For the 4th year students.

ID Number

| R | – | | – | | |
|---|---|---|---|---|---|

1
*Instructions for all participants*

# Instructions for the Experiment on Social Interaction

## Introduction

- During the experiment, please follow the experimenters' directions.
- Talking with others is strictly prohibited.
- Please enter your ID number in the box provided.
- Once everyone is ready, the experimenter will explain the experiment. Please follow along carefully as the experimenter reads through the explanation starting on the next page.
- If during the explanation there is anything you do not understand or if you have other questions, please do not hesitate to raise your hand and let the experimenter know.

# General Outline of the Experiment

- This is an experiment involving a type of monetary transaction.
- The amount of your earnings in the experiment will ultimately vary according to decisions made by each person in the monetary transaction.
- **We will pay everyone today's minimum guaranteed amount of ¥1000 in cash as a show up fee, without exception.** How much extra money you can earn will depend on the results of your monetary transactions during the experiment. We will pay out the extra money earned during the experiment at a later date, as we need time to aggregate the results. The payment schedule will be announced later.

---

- For each monetary transaction, you will be paired with one of the other players.
- One person in each pair will be the ***proposer*** and the other will be the ***recipient*** of the proposal. (A more detailed explanation will follow.)
- In all, the transaction will be repeated four times.
- You will be paired with a different person for each transaction.
- The total reward money you earn today will be calculated from the results of 2 of the 4 transactions.
- Which transactions are used for the calculation will be determined by lottery at the end of the experiment.
- However, it is possible that, depending on the results of the transactions, the reward will be ¥0.
- Whether you can earn extra money above and beyond the ¥1000, the show up fee, will depend on the results of your monetary transactions.
- None of you will find out whom you are paired with, or who made what decisions during or after the experiment.
- Everyone's anonymity will be completely maintained, so please relax and freely make whatever decisions you like.

Next, let us explain the “monetary transaction” in this experiment in more detail.

## 【About The “Monetary Transaction”】

1. The “monetary transaction” takes place between two people, a *proposer* and a *recipient*.

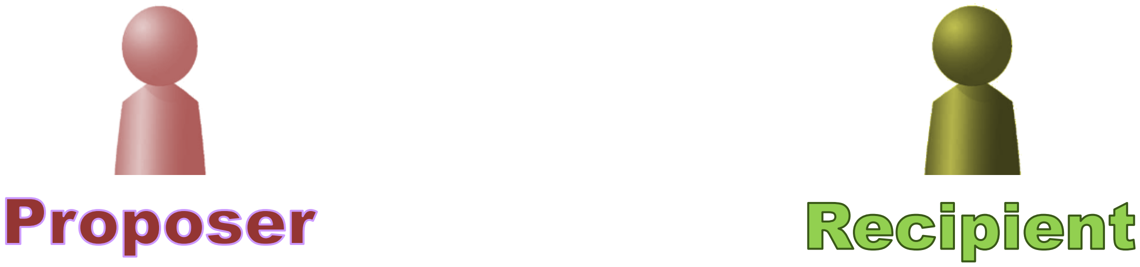


2. The experimenters give the proposer ¥1000 to fund the monetary transaction.

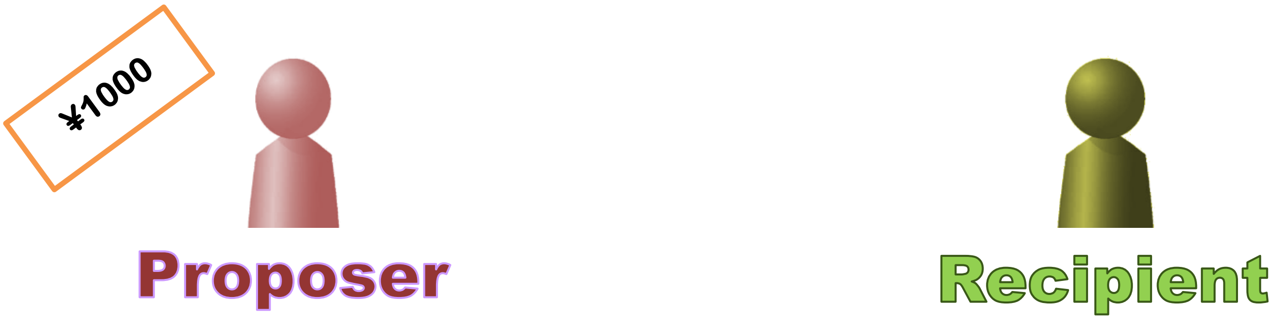


3. The proposer considers how to split the ¥1000 with the recipient and makes a proposal.

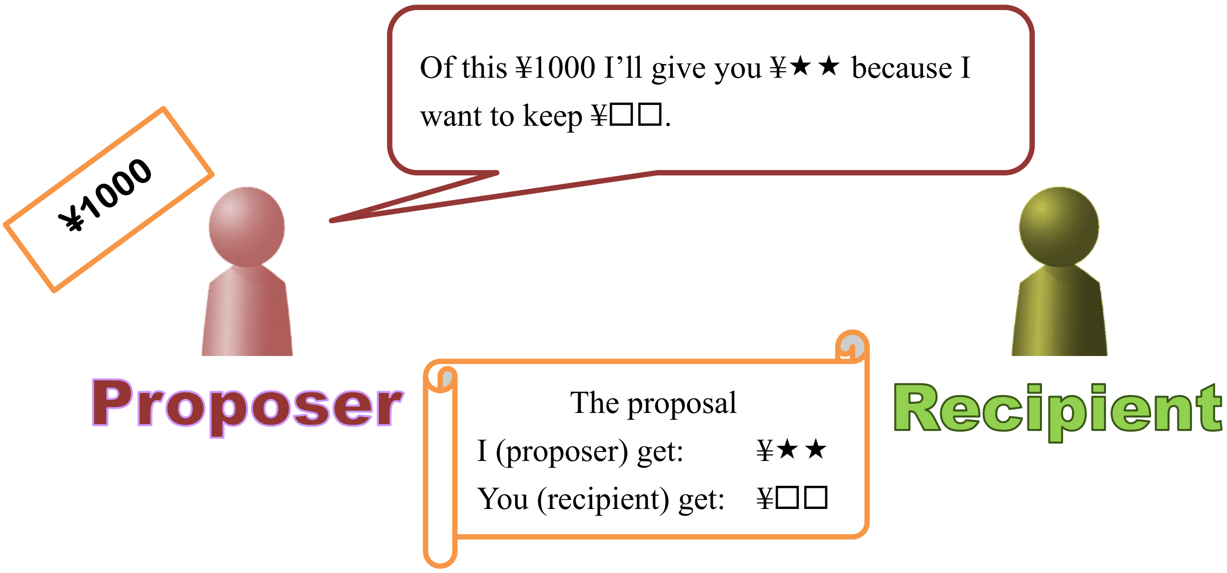

4. The recipient decides to either **accept or reject** the proposed split.

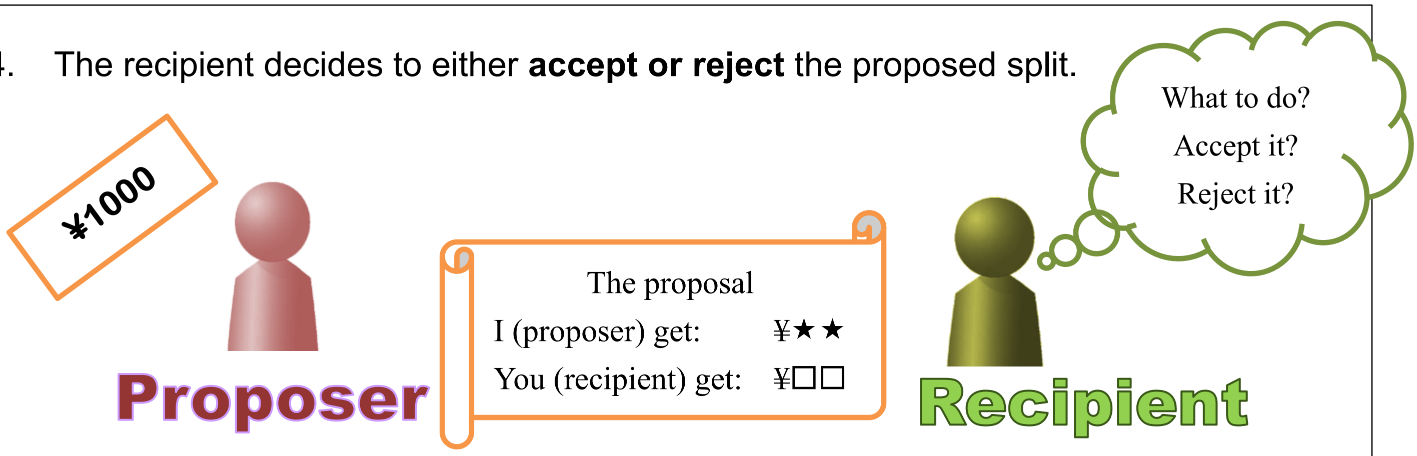


5. If the recipient accepts the proposal ...

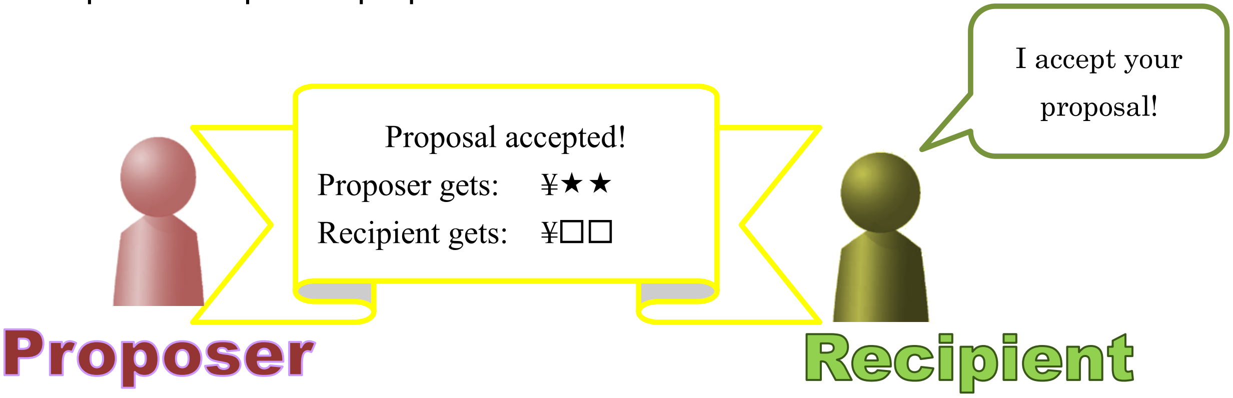


➔ Each receives the proposed amount.

6. If the recipient does not like the proposed amount and rejects it…

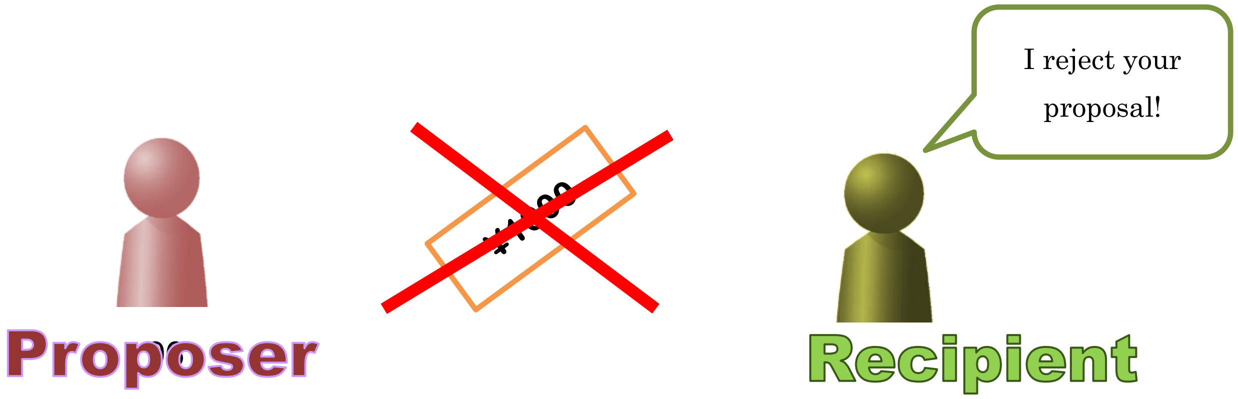


➔ **Both lose all the money and get no reward money for this transaction. (¥0 for each.)**

Next, here are more details about your transaction partners.

## 【About your transaction partners】

1. We will run the monetary transactions four times.
2. **For every transaction you will be paired with a different person and never with the same person twice.**
3. Which player will be the proposer and which player the recipient in each transaction has not been decided yet.
4. At the end of the experiment lotteries will be held to determine which role each person will play and which 2 transactions will be used to calculate the rewards.
5. Therefore, in every monetary transaction…
    1) **First,** everyone acts **as a proposer** and proposes how to split the ¥1000 with the other player.
    2) The proposal is made in increments of ¥100 from ¥0 to ¥1000.
    3) **Next,** everyone acts **as a recipient** and decides whether to accept or reject the other player's (the proposer's) proposal for that transaction.
    4) However, at that point, the actual split proposed by the other player will not be known. Therefore, you will look at a list of possible ¥1000 splits and decide whether you will accept or reject each of them.
6. This procedure will be performed 4 times, with different pairs of players.

**That concludes our explanation of the "monetary transactions" in this experiment. If there is anything that you do not understand, please raise your hand and address your question to the experimenter.**

Enter your ID number here.

| R | – | | – | | |
|---|---|---|---|---|---|

2
*Decision sheet in the first decision for all participants.*

# An Experiment on Monetary Transaction
# Decision Sheets: First transaction

We now begin the experiment.

- You will be given instructions by the experimenter.
- Complete the task as instructed.
- Read this cover page. Do not move on to the next page until you are instructed to do so.

- Your paired partners in these transactions **will be different in each transaction**.
- **You will not know who your transaction partners are** either during or after the experiment.
- Your transaction partner will be determined by lottery at the end of the experiment.
- Further, which 2 of the 4 transactions will be used for actual reward calculation will be determined by lottery. Your earnings for the day will be the total of a minimum guaranteed ¥1000 plus a reward calculated from the results of the 2 transactions selected by lottery.
- We ask that you write your decisions about each transaction on the decision sheets. After all participants have made their decisions, these will be input into a computer to tabulate the results.
- Please wait until the experimenter says to proceed to the next step.
- After completing each transaction, do not turn to the next page. Wait quietly until instructed to continue on to the next page by the experimenter.
- **It is strictly forbidden to try to look at the work of those around you!**

If you have understood the explanation thus far, please wait until instructed to proceed by the experimenter.

**You will be paired with another student from the same school year.**

Please decide what you would do
if you were the ***proposer*.**

For this monetary transaction you have been given ¥1000. How would you like to divide it with the other player? Please write your proposal in the following box.

**Of ¥1000:**

| | |
|---|---|
| **Yourself** | **¥ ________** |
| **Other player** | **¥ ________** |
| **Total** | **¥ 1000** |

Please wait until everyone has finished.

However, please take care others cannot see your proposal.

**You will be paired with another student from the same school year.**

Please decide what you would do
if you were the ***recipient.***

Possible proposals the other player may make are listed below. Please decide whether you would accept or reject each and circle the appropriate response.

| | If the other player proposed the following, what would you do? | | | | **Your decision** (Circle one) | |
|---|---|---|---|---|---|---|
| 1 | To other player | ¥1000, | **To you** | **¥0** | Accept | Reject |
| 2 | To other player | ¥900, | **To you** | **¥100** | Accept | Reject |
| 3 | To other player | ¥800, | **To you** | **¥200** | Accept | Reject |
| 4 | To other player | ¥700, | **To you** | **¥300** | Accept | Reject |
| 5 | To other player | ¥600, | **To you** | **¥400** | Accept | Reject |
| 6 | To other player | ¥500, | **To you** | **¥500** | Accept | Reject |
| 7 | To other player | ¥400, | **To you** | **¥600** | Accept | Reject |
| 8 | To other player | ¥300, | **To you** | **¥700** | Accept | Reject |
| 9 | To other player | ¥200, | **To you** | **¥800** | Accept | Reject |
| 10 | To other player | ¥100, | **To you** | **¥900** | Accept | Reject |
| 11 | To other player | ¥0, | **To you** | **¥1000** | Accept | Reject |

Please ensure you have not missed any responses and close your booklet.

Take care that others cannot see your work.

Enter your ID number here.

| R | – | | – | | |
|---|---|---|---|---|---|

3-1
*Decision sheet in the second decision for 1st year students*

# For 1st-year students

# Decision Sheets:

# Second transaction

The second transaction will now begin.

- In this transaction the other player **<u>will be someone different than in the previous transaction.</u>**

- Do not turn the page until instructed to do so by the experimenter.

- During the experiment **it is strictly forbidden to try to look at the work of those around you.**

Please wait until everyone is ready to begin.

Do not turn the page until everyone is instructed to do so by the experimenter.

**You will be paired with one of the 4th–year students.**

Please decide what you would do if you were the ***proposer*.**

For this monetary transaction you have been given ¥1000. How would you like to divide it with the other player? Please write your proposal in the following box.

**Of ¥1000:**

| | |
|---|---|
| **Yourself** | **¥ ______** |
| **Other player** | **¥ ______** |
| **Total** | **¥ 1000** |

Please wait until everyone has finished.

However, please take care others cannot see your proposal.

# You will be paired with one of the 4th–year students.

Please decide what you would do
if you were the ***recipient.***

Possible proposals the other player may make are listed below. Please decide whether you would accept or reject each and circle the appropriate response.

| | If the other player proposed the following, what would you do? | | | | **Your decision** (Circle one) | |
|---|---|---|---|---|---|---|
| 1 | To other player | ¥1000, | **To you** | **¥0** | Accept | Reject |
| 2 | To other player | ¥900, | **To you** | **¥100** | Accept | Reject |
| 3 | To other player | ¥800, | **To you** | **¥200** | Accept | Reject |
| 4 | To other player | ¥700, | **To you** | **¥300** | Accept | Reject |
| 5 | To other player | ¥600, | **To you** | **¥400** | Accept | Reject |
| 6 | To other player | ¥500, | **To you** | **¥500** | Accept | Reject |
| 7 | To other player | ¥400, | **To you** | **¥600** | Accept | Reject |
| 8 | To other player | ¥300, | **To you** | **¥700** | Accept | Reject |
| 9 | To other player | ¥200, | **To you** | **¥800** | Accept | Reject |
| 10 | To other player | ¥100, | **To you** | **¥900** | Accept | Reject |
| 11 | To other player | ¥0, | **To you** | **¥1000** | Accept | Reject |

Please ensure you have not missed any responses and close your booklet.

Take care that others cannot see your work.

Enter your ID number here.

| R | ― | | ― | | |
|---|---|---|---|---|---|

3-2
*Decision sheet in the second decision for 2nd year students*

# For 2nd -year students

# Decision Sheets:
# Second transaction

The second transaction will now begin.

- In this transaction the other player **will be someone different than in the previous transaction.**
- Do not turn the page until instructed to do so by the experimenter.
- During the experiment **it is strictly forbidden to try to look at the work of those around you.**

Please wait until everyone is ready to begin.

Do not turn the page until everyone is instructed to do so by the experimenter.

**You will be paired with**
**one of the 3rd–year students.**

Please decide what you would do
if you were the ***proposer*****.**

For this monetary transaction you have been given ¥1000. How would you like to divide it with the other player? Please write your proposal in the following box.

| | |
|---|---|
| **Of ¥1000:** | |
| **Yourself** | **¥ ______** |
| **Other player** | **¥ ______** |
| **Total** | **¥ 1000** |

Please wait until everyone has finished.
However, please take care others cannot see your proposal.

## You will be paired with one of the 3rd–year students.

# Please decide what you would do if you were the *recipient.*

Possible proposals the other player may make are listed below. Please decide whether you would accept or reject each and circle the appropriate response.

| | If the other player proposed the following, what would you do? | | | | **Your decision** (Circle one) | |
|---|---|---|---|---|---|---|
| 1 | To other player | ¥1000, | **To you** | **¥0** | Accept | Reject |
| 2 | To other player | ¥900, | **To you** | **¥100** | Accept | Reject |
| 3 | To other player | ¥800, | **To you** | **¥200** | Accept | Reject |
| 4 | To other player | ¥700, | **To you** | **¥300** | Accept | Reject |
| 5 | To other player | ¥600, | **To you** | **¥400** | Accept | Reject |
| 6 | To other player | ¥500, | **To you** | **¥500** | Accept | Reject |
| 7 | To other player | ¥400, | **To you** | **¥600** | Accept | Reject |
| 8 | To other player | ¥300, | **To you** | **¥700** | Accept | Reject |
| 9 | To other player | ¥200, | **To you** | **¥800** | Accept | Reject |
| 10 | To other player | ¥100, | **To you** | **¥900** | Accept | Reject |
| 11 | To other player | ¥0, | **To you** | **¥1000** | Accept | Reject |

Please ensure you have not missed any responses and close your booklet.

Take care that others cannot see your work.

Enter your ID number here.

| R | – | | – | | |
|---|---|---|---|---|---|

3-3
*Decision sheet in the second decision for 3rd year students*

# For 3rd-year students

# Decision Sheets:

# Second transaction

The second transaction will now begin.

- In this transaction the other player **__will be someone different than in the previous transaction.__**
- Do not turn the page until instructed to do so by the experimenter.
- During the experiment **it is strictly forbidden to try to look at the work of those around you.**

Please wait until everyone is ready to begin.

Do not turn the page until everyone is instructed to do so by the experimenter.

**You will be paired with one of the 2nd–year students.**

Please decide what you would do if you were the ***proposer*.**

For this monetary transaction you have been given ¥1000. How would you like to divide it with the other player? Please write your proposal in the following box.

| | |
|---|---|
| **Of ¥1000:** | |
| **Yourself** | **¥ ______** |
| **Other player** | **¥ ______** |
| **Total** | **¥ 1000** |

Please wait until everyone has finished.

However, please take care others cannot see your proposal.

# You will be paired with one of the 2nd–year students.

Please decide what you would do
if you were the ***recipient.***

Possible proposals the other player may make are listed below. Please decide whether you would accept or reject each and circle the appropriate response.

| | If the other player proposed the following, what would you do? | | | | **Your decision** (Circle one) | |
|---|---|---|---|---|---|---|
| 1 | To other player | ¥1000, | **To you** | **¥0** | Accept | Reject |
| 2 | To other player | ¥900, | **To you** | **¥100** | Accept | Reject |
| 3 | To other player | ¥800, | **To you** | **¥200** | Accept | Reject |
| 4 | To other player | ¥700, | **To you** | **¥300** | Accept | Reject |
| 5 | To other player | ¥600, | **To you** | **¥400** | Accept | Reject |
| 6 | To other player | ¥500, | **To you** | **¥500** | Accept | Reject |
| 7 | To other player | ¥400, | **To you** | **¥600** | Accept | Reject |
| 8 | To other player | ¥300, | **To you** | **¥700** | Accept | Reject |
| 9 | To other player | ¥200, | **To you** | **¥800** | Accept | Reject |
| 10 | To other player | ¥100, | **To you** | **¥900** | Accept | Reject |
| 11 | To other player | ¥0, | **To you** | **¥1000** | Accept | Reject |

Please ensure you have not missed any responses and close your booklet.

Take care that others cannot see your work.

Enter your ID number here.

| R | – | | – | | |
|---|---|---|---|---|---|

3-4
*Decision sheet in the second decision for 4th year students*

# For 4th-year students

## Decision Sheets:

## Second transaction

The second transaction will now begin.

- In this transaction the other player **will be someone different than in the previous transaction.**
- Do not turn the page until instructed to do so by the experimenter.
- During the experiment **it is strictly forbidden to try to look at the work of those around you.**

Please wait until everyone is ready to begin.

Do not turn the page until everyone is instructed to do so by the experimenter.

**You will be paired with one of the 1st–year students.**

Please decide what you would do if you were the ***proposer*.**

For this monetary transaction you have been given ¥1000. How would you like to divide it with the other player? Please write your proposal in the following box.

| | |
|---|---|
| **Of ¥1000:** | |
| **Yourself** | **¥ ________** |
| **Other player** | **¥ ________** |
| **Total** | **¥ 1000** |

Please wait until everyone has finished.

However, please take care others cannot see your proposal.

**You will be paired with one of the 1st–year students.**

Please decide what you would do
if you were the ***recipient.***

Possible proposals the other player may make are listed below. Please decide whether you would accept or reject each and circle the appropriate response.

| | If the other player proposed the following, what would you do? | | | | **Your decision** (Circle one) | |
|---|---|---|---|---|---|---|
| 1 | To other player | ¥1000, | **To you** | **¥0** | Accept | Reject |
| 2 | To other player | ¥900, | **To you** | **¥100** | Accept | Reject |
| 3 | To other player | ¥800, | **To you** | **¥200** | Accept | Reject |
| 4 | To other player | ¥700, | **To you** | **¥300** | Accept | Reject |
| 5 | To other player | ¥600, | **To you** | **¥400** | Accept | Reject |
| 6 | To other player | ¥500, | **To you** | **¥500** | Accept | Reject |
| 7 | To other player | ¥400, | **To you** | **¥600** | Accept | Reject |
| 8 | To other player | ¥300, | **To you** | **¥700** | Accept | Reject |
| 9 | To other player | ¥200, | **To you** | **¥800** | Accept | Reject |
| 10 | To other player | ¥100, | **To you** | **¥900** | Accept | Reject |
| 11 | To other player | ¥0, | **To you** | **¥1000** | Accept | Reject |

Please ensure you have not missed any responses and close your booklet.

Take care that others cannot see your work.

↓ Enter your ID number here.

| R | — | | — | | |
|---|---|---|---|---|---|

4-1
*Decision sheet in the third decision for 1st year students*

# For 1st-year students

# Decision Sheets:
# Third transaction

The third transaction will now begin.

- In this transaction the other player **<u>will be someone different than in the previous transaction.</u>**
- Do not turn the page until instructed to do so by the experimenter.
- During the experiment **it is strictly forbidden to try to look at the work of those around you.**

Please wait until everyone is ready to begin.

Do not turn the page until everyone is instructed to do so by the experimenter.

**You will be paired with**
**one of the 3rd–year students.**

Please decide what you would do
if you were the ***proposer*.**

For this monetary transaction you have been given ¥1000. How would you like to divide it with the other player? Please write your proposal in the following box.

| | |
|---|---|
| **Of ¥1000:** | |
| **Yourself** | **¥ ______** |
| **Other player** | **¥ ______** |
| **Total** | **¥ 1000** |

Please wait until everyone has finished.
However, please take care others cannot see your proposal.

# You will be paired with one of the 3rd–year students.

## Please decide what you would do if you were the ***recipient.***

Possible proposals the other player may make are listed below. Please decide whether you would accept or reject each and circle the appropriate response.

| | If the other player proposed the following, what would you do? | | | | **Your decision** (Circle one) | |
|---|---|---|---|---|---|---|
| 1 | To other player | ¥1000, | **To you** | **¥0** | Accept | Reject |
| 2 | To other player | ¥900, | **To you** | **¥100** | Accept | Reject |
| 3 | To other player | ¥800, | **To you** | **¥200** | Accept | Reject |
| 4 | To other player | ¥700, | **To you** | **¥300** | Accept | Reject |
| 5 | To other player | ¥600, | **To you** | **¥400** | Accept | Reject |
| 6 | To other player | ¥500, | **To you** | **¥500** | Accept | Reject |
| 7 | To other player | ¥400, | **To you** | **¥600** | Accept | Reject |
| 8 | To other player | ¥300, | **To you** | **¥700** | Accept | Reject |
| 9 | To other player | ¥200, | **To you** | **¥800** | Accept | Reject |
| 10 | To other player | ¥100, | **To you** | **¥900** | Accept | Reject |
| 11 | To other player | ¥0, | **To you** | **¥1000** | Accept | Reject |

Please ensure you have not missed any responses and close your booklet.

Take care that others cannot see your work.

Enter your ID number here.

| R | – | | – | | |
|---|---|---|---|---|---|

4-2
*Decision sheet in the third decision for 2nd year students*

# For 2nd-year students

## Decision Sheets:
## Third transaction

The third transaction will now begin.

- In this transaction the other player **will be someone different than in the previous transaction.**
- Do not turn the page until instructed to do so by the experimenter.
- During the experiment **it is strictly forbidden to try to look at the work of those around you.**

Please wait until everyone is ready to begin.

Do not turn the page until everyone is instructed to do so by the experimenter.

**You will be paired with one of the 4th–year students.**

Please decide what you would do if you were the ***proposer*.**

For this monetary transaction you have been given ¥1000. How would you like to divide it with the other player? Please write your proposal in the following box.

**Of ¥1000:**

| | |
|---|---|
| **Yourself** | **¥ ______** |
| **Other player** | **¥ ______** |
| **Total** | **¥ 1000** |

Please wait until everyone has finished.

However, please take care others cannot see your proposal.

## You will be paired with one of the 4th–year students.

Please decide what you would do if you were the ***recipient.***

Possible proposals the other player may make are listed below. Please decide whether you would accept or reject each and circle the appropriate response.

| | If the other player proposed the following, what would you do? | | | | **Your decision** (Circle one) | |
|---|---|---|---|---|---|---|
| 1 | To other player | ¥1000, | **To you** | **¥0** | Accept | Reject |
| 2 | To other player | ¥900, | **To you** | **¥100** | Accept | Reject |
| 3 | To other player | ¥800, | **To you** | **¥200** | Accept | Reject |
| 4 | To other player | ¥700, | **To you** | **¥300** | Accept | Reject |
| 5 | To other player | ¥600, | **To you** | **¥400** | Accept | Reject |
| 6 | To other player | ¥500, | **To you** | **¥500** | Accept | Reject |
| 7 | To other player | ¥400, | **To you** | **¥600** | Accept | Reject |
| 8 | To other player | ¥300, | **To you** | **¥700** | Accept | Reject |
| 9 | To other player | ¥200, | **To you** | **¥800** | Accept | Reject |
| 10 | To other player | ¥100, | **To you** | **¥900** | Accept | Reject |
| 11 | To other player | ¥0, | **To you** | **¥1000** | Accept | Reject |

Please ensure you have not missed any responses and close your booklet.

Take care that others cannot see your work.

Enter your ID number here.

| R | — | | — | | |
|---|---|---|---|---|---|

4-3
*Decision sheet in the third decision for 3rd year students*

# For 3rd-year students

## Decision Sheets:

## Third transaction

The third transaction will now begin.

- In this transaction the other player **will be someone different than in the previous transaction.**
- Do not turn the page until instructed to do so by the experimenter.
- During the experiment **it is strictly forbidden to try to look at the work of those around you.**

Please wait until everyone is ready to begin.

Do not turn the page until everyone is instructed to do so by the experimenter.

**You will be paired with one of the 1st–year students.**

Please decide what you would do
if you were the ***proposer*.**

For this monetary transaction you have been given ¥1000. How would you like to divide it with the other player? Please write your proposal in the following box.

| | |
|---|---|
| **Of ¥1000:** | |
| **Yourself** | **¥ ______** |
| **Other player** | **¥ ______** |
| **Total** | **¥ 1000** |

Please wait until everyone has finished.

However, please take care others cannot see your proposal.

# You will be paired with one of the 1st–year students.

Please decide what you would do if you were the ***recipient.***

Possible proposals the other player may make are listed below. Please decide whether you would accept or reject each and circle the appropriate response.

| | If the other player proposed the following, what would you do? | | | | **Your decision** (Circle one) | |
|---|---|---|---|---|---|---|
| 1 | To other player | ¥1000, | **To you** | **¥0** | Accept | Reject |
| 2 | To other player | ¥900, | **To you** | **¥100** | Accept | Reject |
| 3 | To other player | ¥800, | **To you** | **¥200** | Accept | Reject |
| 4 | To other player | ¥700, | **To you** | **¥300** | Accept | Reject |
| 5 | To other player | ¥600, | **To you** | **¥400** | Accept | Reject |
| 6 | To other player | ¥500, | **To you** | **¥500** | Accept | Reject |
| 7 | To other player | ¥400, | **To you** | **¥600** | Accept | Reject |
| 8 | To other player | ¥300, | **To you** | **¥700** | Accept | Reject |
| 9 | To other player | ¥200, | **To you** | **¥800** | Accept | Reject |
| 10 | To other player | ¥100, | **To you** | **¥900** | Accept | Reject |
| 11 | To other player | ¥0, | **To you** | **¥1000** | Accept | Reject |

Please ensure you have not missed any responses and close your booklet.

Take care that others cannot see your work.

Enter your ID number here.

| R | — | | — | | |
|---|---|---|---|---|---|

4-4
*Decision sheet in the third decision for 4th year students*

# For 4th-year students

# Decision Sheets:
# Third transaction

The third transaction will now begin.

- In this transaction the other player **will be someone different than in the previous transaction.**
- Do not turn the page until instructed to do so by the experimenter.
- During the experiment **it is strictly forbidden to try to look at the work of those around you.**

Please wait until everyone is ready to begin.

Do not turn the page until everyone is instructed to do so by the experimenter.

**You will be paired with one of the 2nd–year students.**

Please decide what you would do if you were the ***proposer*.**

For this monetary transaction you have been given ¥1000. How would you like to divide it with the other player? Please write your proposal in the following box.

**Of ¥1000:**

| | |
|---|---|
| **Yourself** | **¥ ______** |
| **Other player** | **¥ ______** |
| **Total** | **¥ 1000** |

Please wait until everyone has finished.

However, please take care others cannot see your proposal.

**You will be paired with one of the 2nd–year students.**

Please decide what you would do if you were the ***recipient.***

Possible proposals the other player may make are listed below. Please decide whether you would accept or reject each and circle the appropriate response.

| | If the other player proposed the following, what would you do? | | | | **Your decision** (Circle one) | |
|---|---|---|---|---|---|---|
| 1 | To other player | ¥1000, | **To you** | **¥0** | Accept | Reject |
| 2 | To other player | ¥900, | **To you** | **¥100** | Accept | Reject |
| 3 | To other player | ¥800, | **To you** | **¥200** | Accept | Reject |
| 4 | To other player | ¥700, | **To you** | **¥300** | Accept | Reject |
| 5 | To other player | ¥600, | **To you** | **¥400** | Accept | Reject |
| 6 | To other player | ¥500, | **To you** | **¥500** | Accept | Reject |
| 7 | To other player | ¥400, | **To you** | **¥600** | Accept | Reject |
| 8 | To other player | ¥300, | **To you** | **¥700** | Accept | Reject |
| 9 | To other player | ¥200, | **To you** | **¥800** | Accept | Reject |
| 10 | To other player | ¥100, | **To you** | **¥900** | Accept | Reject |
| 11 | To other player | ¥0, | **To you** | **¥1000** | Accept | Reject |

Please ensure you have not missed any responses and close your booklet.

Take care that others cannot see your work.

Enter your ID number here.

| R | – | | – | | |
|---|---|---|---|---|---|

5-1
*Decision sheet in the fourth decision for 1st year students*

# For 1st-year students

# Decision Sheets: Fourth transaction

The fourth transaction will now begin.

- In this transaction the other player **will be someone different than in the previous transaction.**
- Do not turn the page until instructed to do so by the experimenter.
- During the experiment **it is strictly forbidden to try to look at the work of those around you.**

Please wait until everyone is ready to begin.

Do not turn the page until everyone is instructed to do so by the experimenter.

**You will be paired with one of the 2nd–year students.**

Please decide what you would do if you were the ***proposer*.**

For this monetary transaction you have been given ¥1000. How would you like to divide it with the other player? Please write your proposal in the following box.

| | |
|---|---|
| **Of ¥1000:** | |
| **Yourself** | **¥ ______** |
| **Other player** | **¥ ______** |
| **Total** | **¥ 1000** |

Please wait until everyone has finished.

However, please take care others cannot see your proposal.

**You will be paired with**
**one of the 2nd–year students.**

Please decide what you would do
if you were the ***recipient.***

Possible proposals the other player may make are listed below. Please decide whether you would accept or reject each and circle the appropriate response.

| | If the other player proposed the following, what would you do? | | | | **Your decision** (Circle one) | |
|---|---|---|---|---|---|---|
| 1 | To other player | ¥1000, | **To you** | **¥0** | Accept | Reject |
| 2 | To other player | ¥900, | **To you** | **¥100** | Accept | Reject |
| 3 | To other player | ¥800, | **To you** | **¥200** | Accept | Reject |
| 4 | To other player | ¥700, | **To you** | **¥300** | Accept | Reject |
| 5 | To other player | ¥600, | **To you** | **¥400** | Accept | Reject |
| 6 | To other player | ¥500, | **To you** | **¥500** | Accept | Reject |
| 7 | To other player | ¥400, | **To you** | **¥600** | Accept | Reject |
| 8 | To other player | ¥300, | **To you** | **¥700** | Accept | Reject |
| 9 | To other player | ¥200, | **To you** | **¥800** | Accept | Reject |
| 10 | To other player | ¥100, | **To you** | **¥900** | Accept | Reject |
| 11 | To other player | ¥0, | **To you** | **¥1000** | Accept | Reject |

Please ensure you have not missed any responses and close your booklet.

Take care that others cannot see your work.

Enter your ID number here.

| R | – | | – | | |
|---|---|---|---|---|---|

5-2
*Decision sheet in the fourth decision for 2nd year students*

# For 2nd-year students

## Decision Sheets:

## Fourth transaction

The fourth transaction will now begin.

- In this transaction the other player **will be someone different than in the previous transaction.**
- Do not turn the page until instructed to do so by the experimenter.
- During the experiment **it is strictly forbidden to try to look at the work of those around you.**

Please wait until everyone is ready to begin.

Do not turn the page until everyone is instructed to do so by the experimenter.

**You will be paired with**
**one of the 1st–year students.**

Please decide what you would do
if you were the ***proposer*****.**

For this monetary transaction you have been given ¥1000. How would you like to divide it with the other player? Please write your proposal in the following box.

**Of ¥1000:**

| | |
|---|---|
| **Yourself** | **¥ ________** |
| **Other player** | **¥ ________** |
| **Total** | **¥ 1000** |

Please wait until everyone has finished.
However, please take care others cannot see your proposal.

## You will be paired with one of the 1st–year students.

Please decide what you would do if you were the ***recipient.***

Possible proposals the other player may make are listed below. Please decide whether you would accept or reject each and circle the appropriate response.

| | If the other player proposed the following, what would you do? | **Your decision** (Circle one) | |
|---|---|---|---|
| 1 | To other player ¥1000, **To you ¥0** | Accept | Reject |
| 2 | To other player ¥900, **To you ¥100** | Accept | Reject |
| 3 | To other player ¥800, **To you ¥200** | Accept | Reject |
| 4 | To other player ¥700, **To you ¥300** | Accept | Reject |
| 5 | To other player ¥600, **To you ¥400** | Accept | Reject |
| 6 | To other player ¥500, **To you ¥500** | Accept | Reject |
| 7 | To other player ¥400, **To you ¥600** | Accept | Reject |
| 8 | To other player ¥300, **To you ¥700** | Accept | Reject |
| 9 | To other player ¥200, **To you ¥800** | Accept | Reject |
| 10 | To other player ¥100, **To you ¥900** | Accept | Reject |
| 11 | To other player ¥0, **To you ¥1000** | Accept | Reject |

Please ensure you have not missed any responses and close your booklet.

Take care that others cannot see your work.

Enter your ID number here.

| R | – | | – | | |
|---|---|---|---|---|---|

5-3
*Decision sheet in the fourth decision for 3rd year students*

# For 3rd-year students

# Decision Sheets:
# Fourth transaction

The fourth transaction will now begin.

- In this transaction the other player **will be someone different than in the previous transaction.**
- Do not turn the page until instructed to do so by the experimenter.
- During the experiment **it is strictly forbidden to try to look at the work of those around you.**

Please wait until everyone is ready to begin.

Do not turn the page until everyone is instructed to do so by the experimenter.

**You will be paired with one of the 4th–year students.**

Please decide what you would do if you were the ***proposer*.**

For this monetary transaction you have been given ¥1000. How would you like to divide it with the other player? Please write your proposal in the following box.

**Of ¥1000:**

| | |
|---|---|
| **Yourself** | **¥ ______** |
| **Other player** | **¥ ______** |
| **Total** | **¥ 1000** |

Please wait until everyone has finished.

However, please take care others cannot see your proposal.

# You will be paired with one of the 4th–year students.

Please decide what you would do if you were the ***recipient.***

Possible proposals the other player may make are listed below. Please decide whether you would accept or reject each and circle the appropriate response.

| | If the other player proposed the following, what would you do? | | | | **Your decision** (Circle one) | |
|---|---|---|---|---|---|---|
| 1 | To other player | ¥1000, | **To you** | **¥0** | Accept | Reject |
| 2 | To other player | ¥900, | **To you** | **¥100** | Accept | Reject |
| 3 | To other player | ¥800, | **To you** | **¥200** | Accept | Reject |
| 4 | To other player | ¥700, | **To you** | **¥300** | Accept | Reject |
| 5 | To other player | ¥600, | **To you** | **¥400** | Accept | Reject |
| 6 | To other player | ¥500, | **To you** | **¥500** | Accept | Reject |
| 7 | To other player | ¥400, | **To you** | **¥600** | Accept | Reject |
| 8 | To other player | ¥300, | **To you** | **¥700** | Accept | Reject |
| 9 | To other player | ¥200, | **To you** | **¥800** | Accept | Reject |
| 10 | To other player | ¥100, | **To you** | **¥900** | Accept | Reject |
| 11 | To other player | ¥0, | **To you** | **¥1000** | Accept | Reject |

Please ensure you have not missed any responses and close your booklet.

Take care that others cannot see your work.

Enter your ID number here.

| R | – | | – | | |
|---|---|---|---|---|---|

5-4
*Decision sheet in the fourth decision for 4th year students*

# For 4th-year students

# Decision Sheets:
# Fourth transaction

The fourth transaction will now begin.

- In this transaction the other player **will be someone different than in the previous transaction.**
- Do not turn the page until instructed to do so by the experimenter.
- During the experiment **it is strictly forbidden to try to look at the work of those around you.**

Please wait until everyone is ready to begin.

Do not turn the page until everyone is instructed to do so by the experimenter.

**You will be paired with one of the 3rd–year students.**

Please decide what you would do if you were the ***proposer*.**

For this monetary transaction you have been given ¥1000. How would you like to divide it with the other player? Please write your proposal in the following box.

**Of ¥1000:**

| | |
|---|---|
| **Yourself** | **¥ ________** |
| **Other player** | **¥ ________** |
| **Total** | **¥ 1000** |

Please wait until everyone has finished.

However, please take care others cannot see your proposal.

# You will be paired with one of the 3rd–year students.

## Please decide what you would do if you were the ***recipient.***

Possible proposals the other player may make are listed below. Please decide whether you would accept or reject each and circle the appropriate response.

| | If the other player proposed the following, what would you do? | | | | **Your decision** (Circle one) | |
|---|---|---|---|---|---|---|
| 1 | To other player | ¥1000, | **To you** | **¥0** | Accept | Reject |
| 2 | To other player | ¥900, | **To you** | **¥100** | Accept | Reject |
| 3 | To other player | ¥800, | **To you** | **¥200** | Accept | Reject |
| 4 | To other player | ¥700, | **To you** | **¥300** | Accept | Reject |
| 5 | To other player | ¥600, | **To you** | **¥400** | Accept | Reject |
| 6 | To other player | ¥500, | **To you** | **¥500** | Accept | Reject |
| 7 | To other player | ¥400, | **To you** | **¥600** | Accept | Reject |
| 8 | To other player | ¥300, | **To you** | **¥700** | Accept | Reject |
| 9 | To other player | ¥200, | **To you** | **¥800** | Accept | Reject |
| 10 | To other player | ¥100, | **To you** | **¥900** | Accept | Reject |
| 11 | To other player | ¥0, | **To you** | **¥1000** | Accept | Reject |

Please ensure you have not missed any responses and close your booklet.

Take care that others cannot see your work.
